# Supplementary material for: Mutant huntingtin induces neuronal apoptosis via derepressing the non-canonical poly(A) polymerase PAPD5
Source: Nat Commun. 2025 Apr 9;16:3307. doi: 10.1038/s41467-025-58618-4 (PMC11982267; doi:10.1038/s41467-025-58618-4)
Supplement: Supplementary file 1 — Supplementary information [file 41467_2025_58618_MOESM1_ESM.docx]

**Supplementary information**

**Mutant huntingtin induces neuronal apoptosis via derepressing the non-canonical poly(A) polymerase PAPD5**

Zhefan Stephen Chen^1,2,#^, Shaohong Isaac Peng^1,#^, Lok I Leong^1^, Terence Gall-Duncan^3,4^, Nathan Siu Jun Wong^1^, Tsz Ho Li^1^, Xiao Lin^1^, Yuming Wei^1^, Alex Chun Koon^1^, Junzhe Huang^5^, Jacquelyne Ka-Li Sun^1^, Clinton Turner^6^, Lynette Tippett^7,8^, Maurice A. Curtis^8,9^, Richard L.M. Faull^7,9^, Kin Ming Kwan^1,10,11^, Hei-Man Chow^1,2^, Ho Ko^2,5,12^, Ting-Fung Chan^1,10^, Kevin Talbot^13,14^, Christopher E. Pearson^3,15^ and Ho Yin Edwin Chan^1,2,*^

^1^School of Life Sciences, The Chinese University of Hong Kong, Shatin, N.T., Hong Kong SAR, China

^2^Gerald Choa Neuroscience Institute, The Chinese University of Hong Kong, Shatin, N.T., Hong Kong SAR, China

^3^Genetics & Genome Biology, The Hospital for Sick Children, Toronto, ON, Canada

^4^Molecular Genetics, University of Toronto, Toronto, ON, Canada

^5^Division of Neurology, Department of Medicine and Therapeutics, Faculty of Medicine, The Chinese University of Hong Kong, Shatin, N.T., Hong Kong SAR, China

^6^Anatomical Pathology, LabPlus, Auckland City Hospital, Auckland, New Zealand

^7^School of Psychology, University of Auckland, Auckland, New Zealand

^8^University Research Centre for Brain Research, University of Auckland, Auckland, New Zealand

^9^Anatomy and Medical Imaging, University of Auckland, Auckland, New Zealand

^10^State Key Laboratory of Agrobiotechnology (CUHK), The Chinese University of Hong Kong, Shatin, N.T., Hong Kong SAR, China

^11^Centre for Cell and Developmental Biology, The Chinese University of Hong Kong, Shatin, N.T., Hong Kong SAR, China

^12^Li Ka Shing Institute of Health Sciences, Faculty of Medicine, The Chinese University of Hong Kong, Shatin, N.T., Hong Kong SAR, China

^13^Oxford Motor Neuron Disease Centre, Nuffield Department of Clinical Neurosciences, John Radcliffe Hospital, University of Oxford, Oxford OX3 9DU, UK

^14^Kavli Institute for Nanoscience Discovery, University of Oxford, Dorothy Crowfoot Hodgkin Building, South Parks Road, Oxford OX1 3QU, UK

^15^Structural Genomics Consortium, University of Toronto, Toronto, ON, Canada

^#^These authors contributed equally to this work

* Correspondence: [hyechan@cuhk.edu.hk](mailto:hyechan@cuhk.edu.hk) (H.Y.E.C.)

**
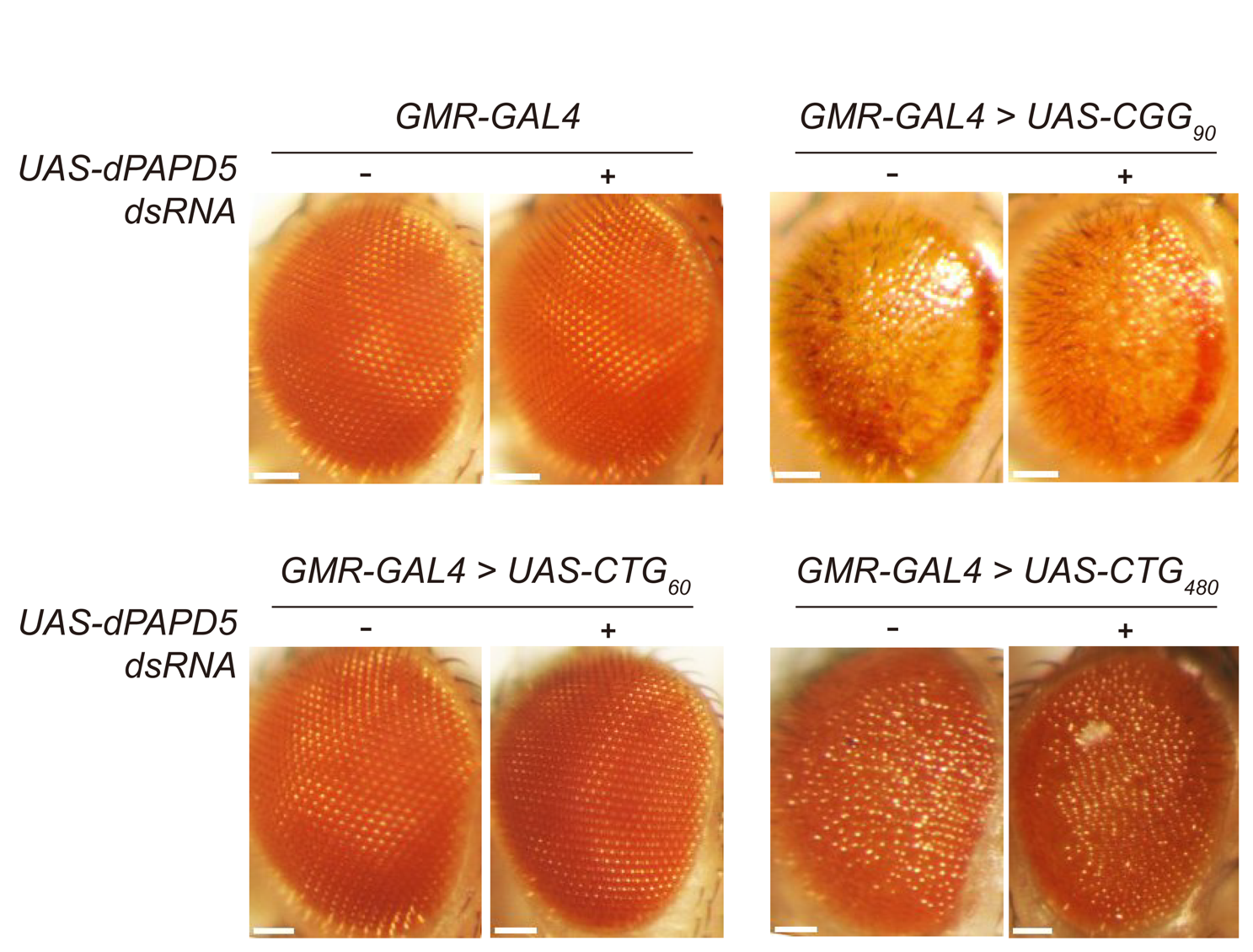
**

**Supplementary Fig. 1 Knockdown of *dPAPD5* does not rescue the external eye degeneration induced by mutant *CGG*- or *CUG*-repeat RNA in *Drosophila*.**

Knockdown of *dPAPD5* did not alter the external eye degeneration in *CGG_90_* and *CTG_480_* flies. Knockdown of *dPAPD5* did not cause dominant eye degeneration in driver control and *CTG_60_* flies. The genotypes of flies are listed in Supplementary Table 5. Scale bars: 20 µm. *n* = 3 biologically independent experiments.

**
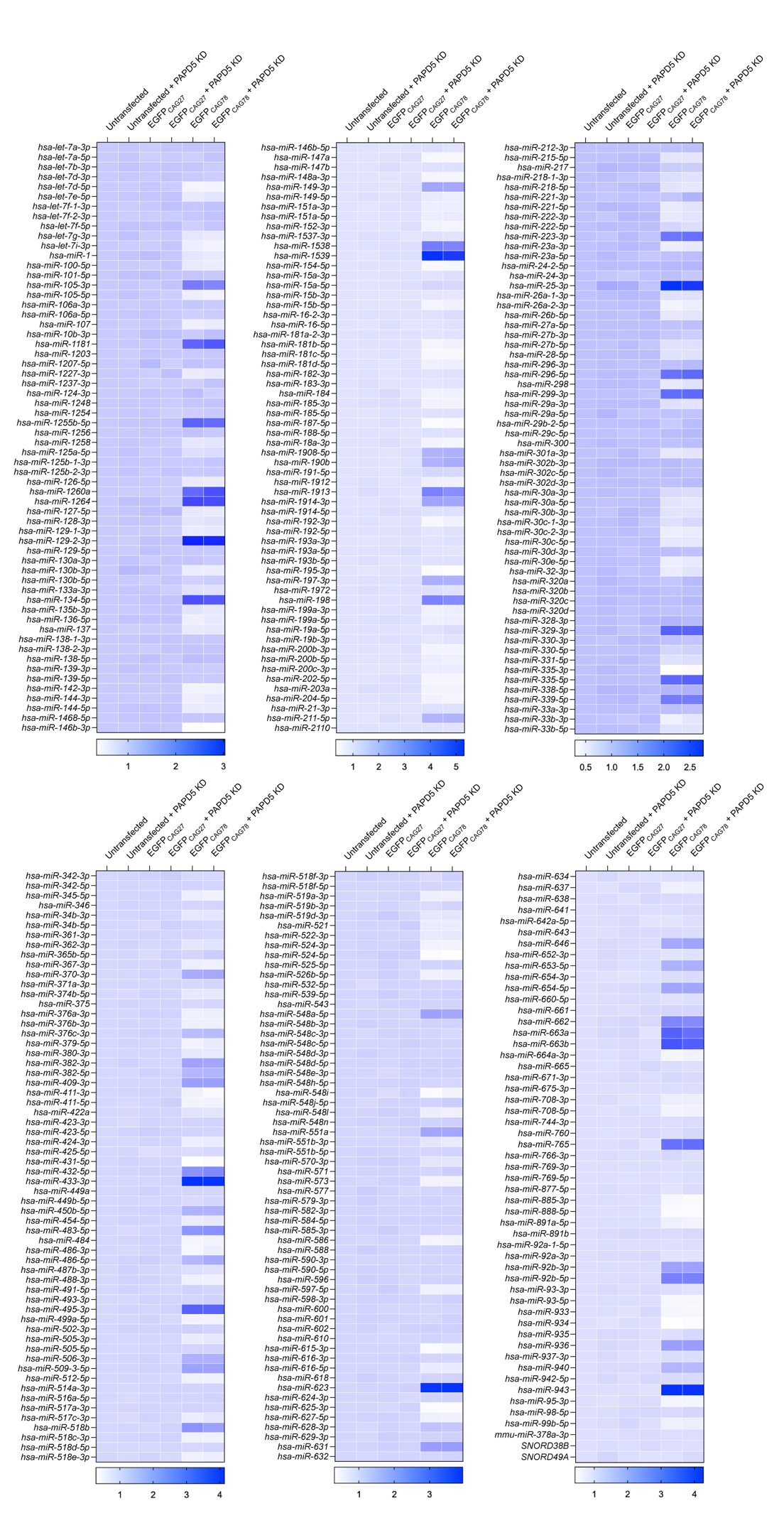
**

**Supplementary Fig. 2 Heat map analysis of the miRNA array data.**

Summary of the miRNAs that fall into the following two categories: 1) did not show dysregulation in *EGFP_CAG78_*-expressing cells; and 2) showed dysregulation in *EGFP_CAG78_*-expressing cells, but such dysregulation was not restored upon knockdown of *PAPD5*.

**
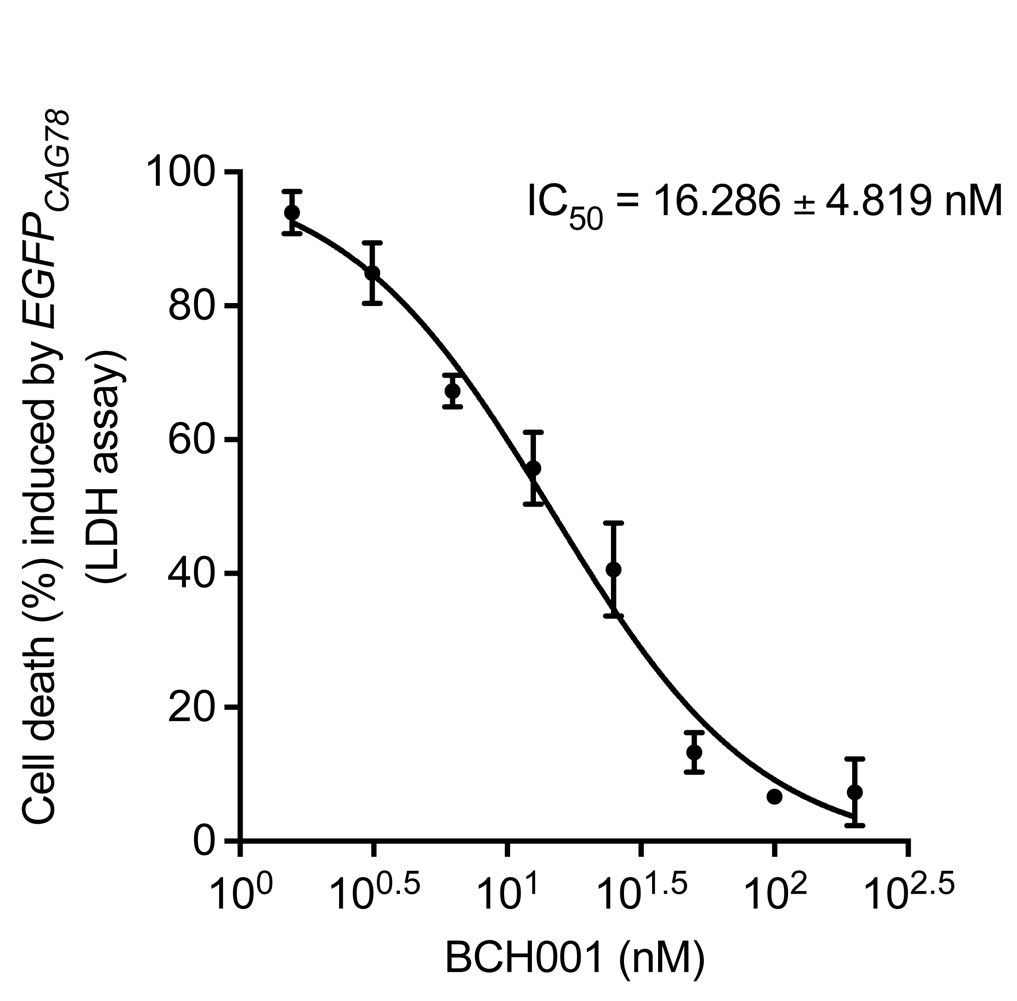
**

**Supplementary Fig. 3 BCH001 rescues mutant CAG RNA-induced cell death.**

BCH001 suppressed *EGFP_CAG78_*-induced cell death with an IC_50_ value of 16.29 nM. *n* = 3 biologically independent experiments. Data is presented as mean ± S.E.M. Source data are provided as a Source Data file.

**
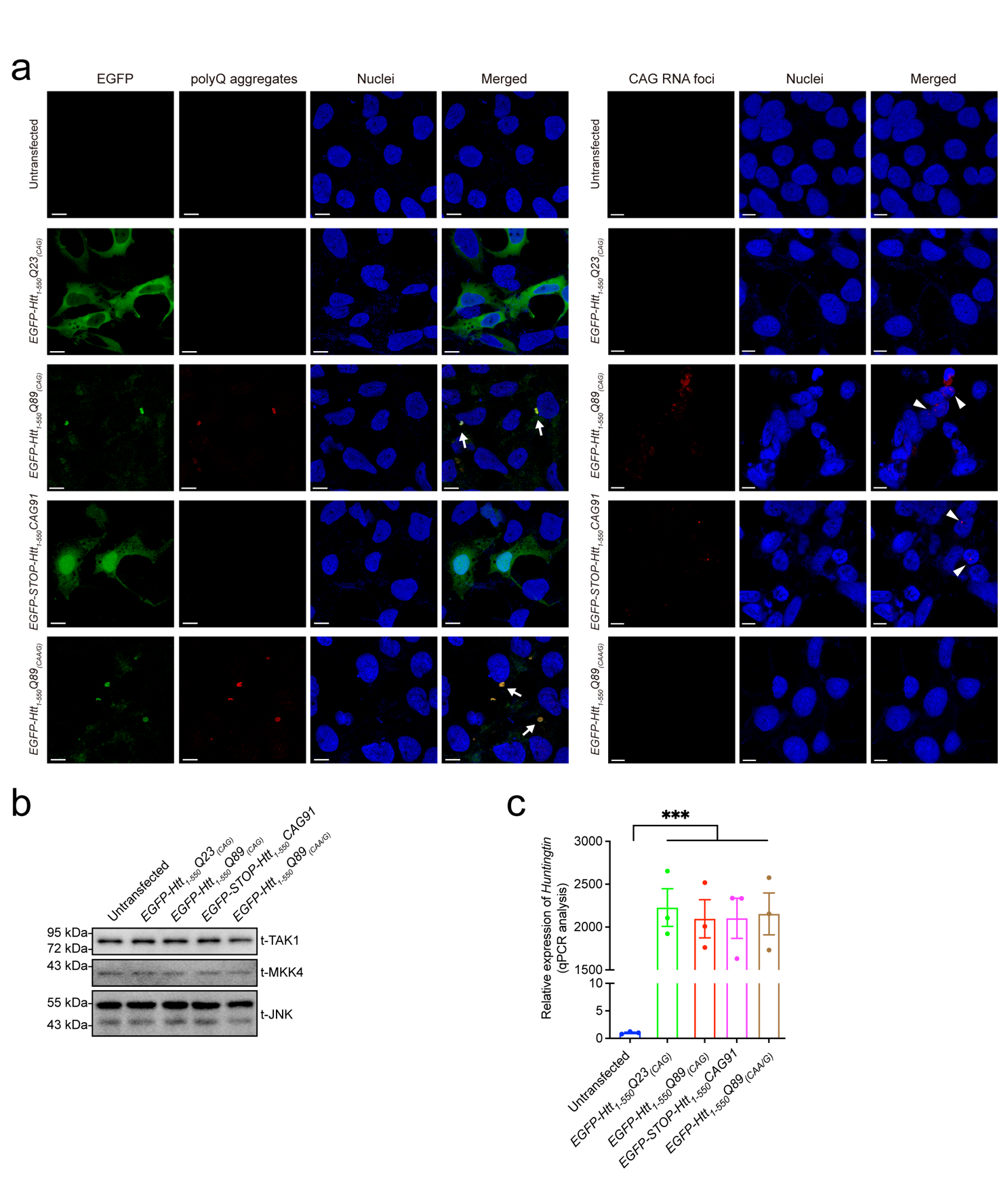
**

**Supplementary Fig. 4 Data related to main Fig. 8.**

**a** Expression of *EGFP-Htt_1-550_Q89_(CAG)_* led to the production of both polyQ protein aggregates and CAG RNA foci. The CAG RNA foci, but not polyQ protein aggregates, was detected in *EGFP-STOP-Htt_1-550_CAG91*-expressing cells. The polyQ protein aggregates, but not CAG RNA foci, were detected in *EGFP-Htt_1-550_Q89_(CAA/G)_*-expressing cells. Neither polyQ protein aggregates nor CAG RNA foci were detected in *EGFP-Htt_1-550_Q23_(CAG)_*-expressing cells. The arrows indicate polyQ protein aggregates, while the arrowheads indicate CAG RNA foci. Cell nuclei (blue) were stained with Hoechst 33342. Scale bars: 10 μm. **b** Representative t-TAK1, t-MKK4 and t-JNK blots of the main Fig. 8j. **c** The expression of *Htt_1-550_* transcript was comparable among different Htt constructs-transfected cells. Statistical analysis was performed using one-way ANOVA followed by *post hoc* Tukey’s test. The exact *P* values are listed in Supplementary Table 6. *n* = 3 biologically independent experiments. Data is presented as mean ± S.E.M. in panel 4c. Source data are provided as a Source Data file.

**
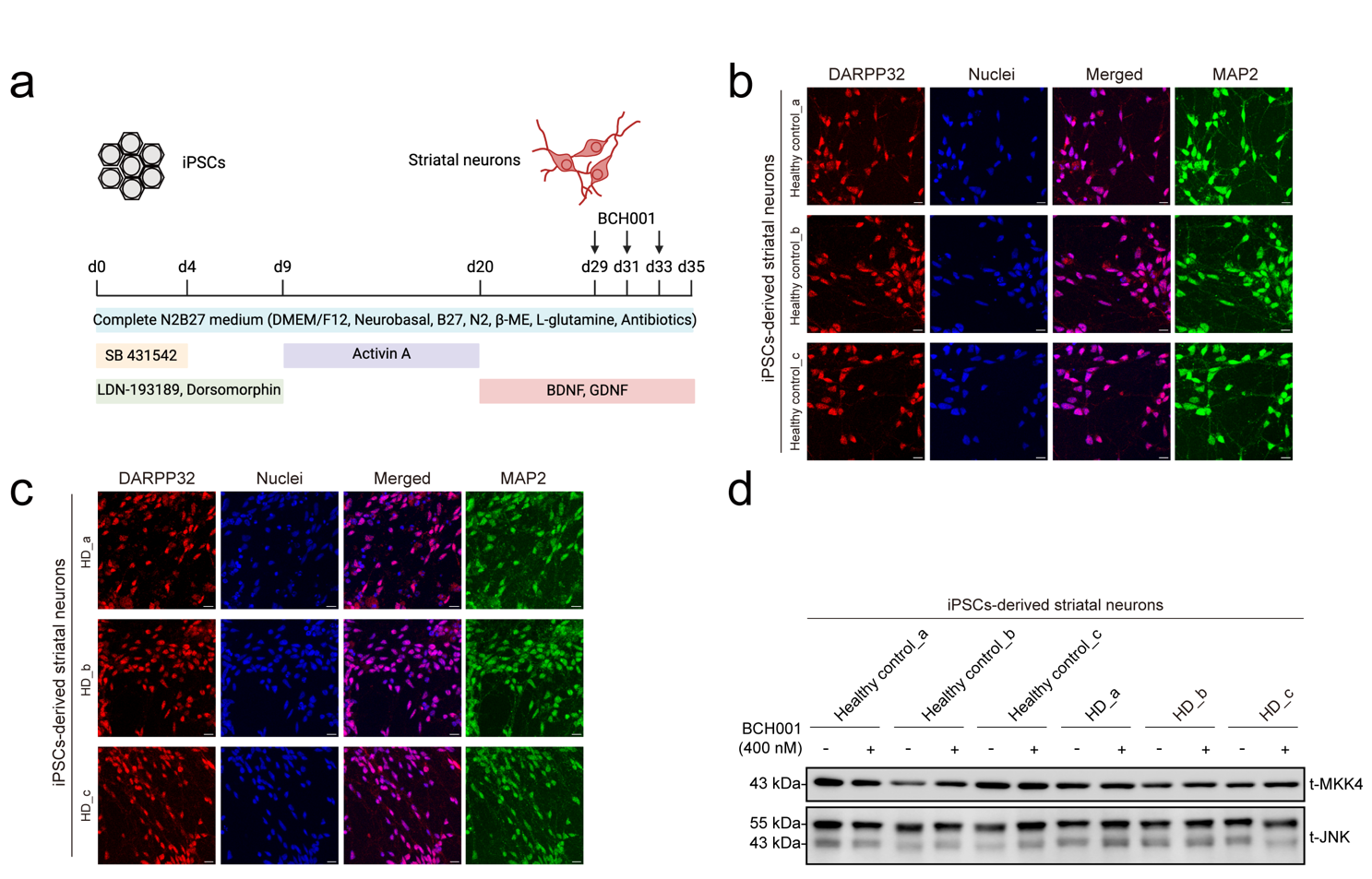
**

**Supplementary Fig. 5 Data related to main Fig. 9.**

**a** Schematic representation of the protocol used for the differentiation of iPSCs into striatal neurons. Created in BioRender. Chen, S. (2025) <https://BioRender.com/j48c711>. **b-c** Representative images of the DARPP32 expression in healthy control (**b**) and HD (**c**) iPSCs-derived striatal neurons. MAP2 labels the cell soma and neurites. Cell nuclei (blue) were stained with Hoechst 33342. Scale bars: 10 μm. **d** Representative t-MKK4 and t-JNK blots of the main Fig. 9e. *n* = 3 biologically independent experiments. Source data are provided as a Source Data file.


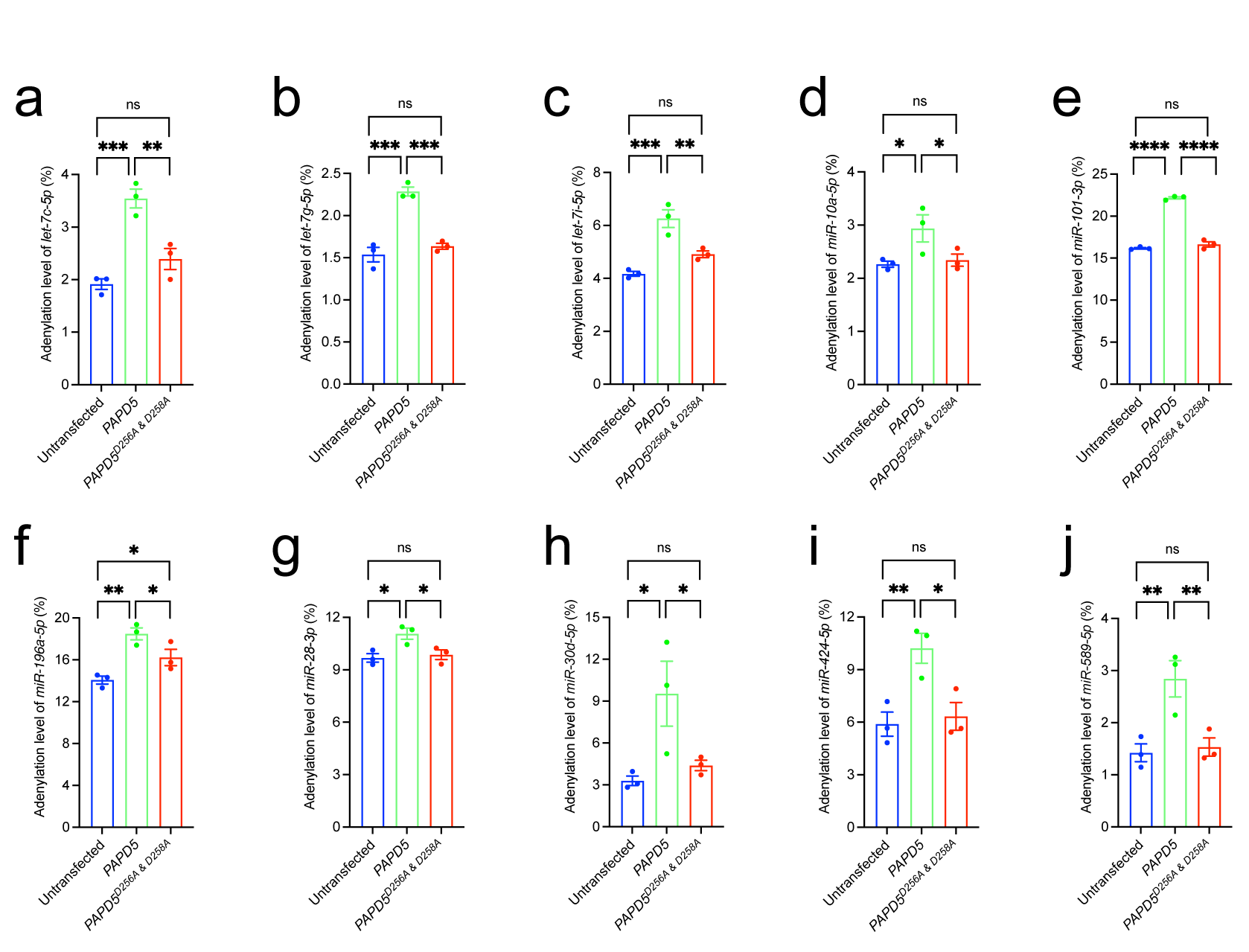


**Supplementary Fig. 6 The catalytic function of PAPD5 is required for regulating the adenylation of a subset of miRNAs.**

**a-j** The adenylation levels of miRNAs, including *let-7c-5p* (**a**), *let-7g-5p* (**b**), *let-7i-5p* (**c**), *miR-10a-5p* (**d**), *miR-101-3p* (**e**), *miR-196a-5p* (**f**), *miR-28-3p* (**g**), *miR-30d-5p* (**h**), *miR-424-5p* (**i**) and *miR-589-5p* (**j**), were significantly induced in PAPD5-, but not PAPD5^D256A & D258A^-overexpressing cells. Statistical analysis was performed using one-way ANOVA followed by *post hoc* Fisher's LSD test. The exact *P* values are listed in Supplementary Table 6. *n* = 3 biologically independent experiments. Data is presented as mean ± S.E.M. in panels 6a-j. Source data are provided in Supplementary Data 2.

**Supplementary Table 1. The inserted DNA sequence of different Htt constructs**

| TGA: Stop codon  AGATCT: *Bgl*II  GGTACC: *Kpn*I |
| --- |
| **Htt_1-550_Q23_(CAG)_ nucleotide sequence:**  AGATCTATGGCGACCCTGGAAAAGCTGATGAAGGCCTTCGAGTCCCTCAAGTCCTTCCAGCAGCAGCAGCAGCAGCAGCAGCAGCAGCAGCAGCAGCAGCAGCAGCAGCAGCAGCAGCAGCAGCAGCAACAGCCGCCACCGCCGCCGCCGCCGCCGCCGCCTCCTCAGCTTCCTCAGCCGCCGCCGCAGGCACAGCCGCTGCTGCCTCAGCCGCAGCCGCCCCCGCCGCCGCCCCCGCCGCCACCCGGCCCGGCTGTGGCTGAGGAGCCGCTGCACCGACCAAAGAAAGAACTTTCAGCTACCAAGAAAGACCGTGTGAATCATTGTCTGACAATATGTGAAAACATAGTGGCACAGTCTGTCAGAAATTCTCCAGAATTTCAGAAACTTCTGGGCATCGCTATGGAACTTTTTCTGCTGTGCAGTGATGACGCAGAGTCAGATGTCAGGATGGTGGCTGACGAATGCCTCAACAAAGTTATCAAAGCTTTGATGGATTCTAATCTTCCAAGGTTACAGCTCGAGCTCTATAAGGAAATTAAAAAGAATGGTGCCCCTCGGAGTTTGCGTGCTGCCCTGTGGAGGTTTGCTGAGCTGGCTCACCTGGTTCGGCCTCAGAAATGCAGGCCTTACCTGGTGAACCTTCTGCCGTGCCTGACTCGAACAAGCAAGAGACCCGAAGAATCAGTCCAGGAGACCTTGGCTGCAGCTGTTCCCAAAATTATGGCTTCTTTTGGCAATTTTGCAAATGACAATGAAATTAAGGTTTTGTTAAAGGCCTTCATAGCGAACCTGAAGTCAAGCTCCCCCACCATTCGGCGGACAGCGGCTGGATCAGCAGTGAGCATCTGCCAGCACTCAAGAAGGACACAATATTTCTATAGTTGGCTACTAAATGTGCTCTTAGGCTTACTCGTTCCTGTCGAGGATGAACACTCCACTCTGCTGATTCTTGGCGTGCTGCTCACCCTGAGGTATTTGGTGCCCTTGCTGCAGCAGCAGGTCAAGGACACAAGCCTGAAAGGCAGCTTCGGAGTGACAAGGAAAGAAATGGAAGTCTCTCCTTCTGCAGAGCAGCTTGTCCAGGTTTATGAACTGACGTTACATCATACACAGCACCAAGACCACAATGTTGTGACCGGAGCCCTGGAGCTGTTGCAGCAGCTCTTCAGAACGCCTCCACCCGAGCTTCTGCAAACCCTGACCGCAGTCGGGGGCATTGGGCAGCTCACCGCTGCTAAGGAGGAGTCTGGTGGCCGAAGCCGTAGTGGGAGTATTGTGGAACTTATAGCTGGAGGGGGTTCCTCATGCAGCCCTGTCCTTTCAAGAAAACAAAAAGGCAAAGTGCTCTTAGGAGAAGAAGAAGCCTTGGAGGATGACTCTGAATCGAGATCGGATGTCAGCAGCTCTGCCTTAACAGCCTCAGTGAAGGATGAGATCAGTGGAGAGCTGGCTGCTTCTTCAGGGGTTTCCACTCCAGGGTCAGCAGGTCATGACATCATCACAGAACAGCCACGGTCACAGCACACACTGCAGGCGGACTCAGTGGATCTGGCCAGCTGTGACTTGACAAGCTCTGCCACTGATGGGGATGAGGAGGATATCTTGAGCCACAGCTCCAGCCAGGTCAGCGCCGTCCCATCTGACCCTGCCATGGACCTGTGAGGTACC |
| **Htt_1-550_Q89_(CAG)_ nucleotide sequence:**  AGATCTATGGCGACCCTGGAAAAGCTGATGAAGGCCTTCGAGTCCCTCAAGTCCTTCCAGCAGCAGCAGCAGCAGCAGCAGCAGCAGCAGCAGCAGCAGCAGCAGCAGCAGCAGCAGCAGCAGCAGCAGCAGCAGCAGCAGCAGCAGCAGCAGCAGCAGCAGCAGCAGCAGCAGCAGCAGCAGCAGCAGCAGCAGCAGCAGCAGCAGCAGCAGCAGCAGCAGCAGCAGCAGCAGCAGCAGCAGCAGCAGCAGCAGCAGCAGCAGCAGCAGCAGCAGCAGCAGCAGCAGCAGCAGCAGCAGCAGCAGCAGCAGCAGCAGCAGCAGCAACAGCCGCCACCGCCGCCGCCGCCGCCGCCGCCTCCTCAGCTTCCTCAGCCGCCGCCGCAGGCACAGCCGCTGCTGCCTCAGCCGCAGCCGCCCCCGCCGCCGCCCCCGCCGCCACCCGGCCCGGCTGTGGCTGAGGAGCCGCTGCACCGACCAAAGAAAGAACTTTCAGCTACCAAGAAAGACCGTGTGAATCATTGTCTGACAATATGTGAAAACATAGTGGCACAGTCTGTCAGAAATTCTCCAGAATTTCAGAAACTTCTGGGCATCGCTATGGAACTTTTTCTGCTGTGCAGTGATGACGCAGAGTCAGATGTCAGGATGGTGGCTGACGAATGCCTCAACAAAGTTATCAAAGCTTTGATGGATTCTAATCTTCCAAGGTTACAGCTCGAGCTCTATAAGGAAATTAAAAAGAATGGTGCCCCTCGGAGTTTGCGTGCTGCCCTGTGGAGGTTTGCTGAGCTGGCTCACCTGGTTCGGCCTCAGAAATGCAGGCCTTACCTGGTGAACCTTCTGCCGTGCCTGACTCGAACAAGCAAGAGACCCGAAGAATCAGTCCAGGAGACCTTGGCTGCAGCTGTTCCCAAAATTATGGCTTCTTTTGGCAATTTTGCAAATGACAATGAAATTAAGGTTTTGTTAAAGGCCTTCATAGCGAACCTGAAGTCAAGCTCCCCCACCATTCGGCGGACAGCGGCTGGATCAGCAGTGAGCATCTGCCAGCACTCAAGAAGGACACAATATTTCTATAGTTGGCTACTAAATGTGCTCTTAGGCTTACTCGTTCCTGTCGAGGATGAACACTCCACTCTGCTGATTCTTGGCGTGCTGCTCACCCTGAGGTATTTGGTGCCCTTGCTGCAGCAGCAGGTCAAGGACACAAGCCTGAAAGGCAGCTTCGGAGTGACAAGGAAAGAAATGGAAGTCTCTCCTTCTGCAGAGCAGCTTGTCCAGGTTTATGAACTGACGTTACATCATACACAGCACCAAGACCACAATGTTGTGACCGGAGCCCTGGAGCTGTTGCAGCAGCTCTTCAGAACGCCTCCACCCGAGCTTCTGCAAACCCTGACCGCAGTCGGGGGCATTGGGCAGCTCACCGCTGCTAAGGAGGAGTCTGGTGGCCGAAGCCGTAGTGGGAGTATTGTGGAACTTATAGCTGGAGGGGGTTCCTCATGCAGCCCTGTCCTTTCAAGAAAACAAAAAGGCAAAGTGCTCTTAGGAGAAGAAGAAGCCTTGGAGGATGACTCTGAATCGAGATCGGATGTCAGCAGCTCTGCCTTAACAGCCTCAGTGAAGGATGAGATCAGTGGAGAGCTGGCTGCTTCTTCAGGGGTTTCCACTCCAGGGTCAGCAGGTCATGACATCATCACAGAACAGCCACGGTCACAGCACACACTGCAGGCGGACTCAGTGGATCTGGCCAGCTGTGACTTGACAAGCTCTGCCACTGATGGGGATGAGGAGGATATCTTGAGCCACAGCTCCAGCCAGGTCAGCGCCGTCCCATCTGACCCTGCCATGGACCTGTGAGGTACC |
| **STOP-Htt_1-550_CAG91 nucleotide sequence (**GTGAATAACTAG: **tandem stop codons):**  AGATCTGTGAATAACTAGATGGCGACCCTGGAAAAGCTGATGAAGGCCTTCGAGTCCCTCAAGTCCTTCCAGCAGCAGCAGCAGCAGCAGCAGCAGCAGCAGCAGCAGCAGCAGCAGCAGCAGCAGCAGCAGCAGCAGCAGCAGCAGCAGCAGCAGCAGCAGCAGCAGCAGCAGCAGCAGCAGCAGCAGCAGCAGCAGCAGCAGCAGCAGCAGCAGCAGCAGCAGCAGCAGCAGCAGCAGCAGCAGCAGCAGCAGCAGCAGCAGCAGCAGCAGCAGCAGCAGCAGCAGCAGCAGCAGCAGCAGCAGCAGCAGCAGCAGCAGCAGCAGCAGCAGCAGCAGCAGCAACAGCCGCCACCGCCGCCGCCGCCGCCGCCGCCTCCTCAGCTTCCTCAGCCGCCGCCGCAGGCACAGCCGCTGCTGCCTCAGCCGCAGCCGCCCCCGCCGCCGCCCCCGCCGCCACCCGGCCCGGCTGTGGCTGAGGAGCCGCTGCACCGACCAAAGAAAGAACTTTCAGCTACCAAGAAAGACCGTGTGAATCATTGTCTGACAATATGTGAAAACATAGTGGCACAGTCTGTCAGAAATTCTCCAGAATTTCAGAAACTTCTGGGCATCGCTATGGAACTTTTTCTGCTGTGCAGTGATGACGCAGAGTCAGATGTCAGGATGGTGGCTGACGAATGCCTCAACAAAGTTATCAAAGCTTTGATGGATTCTAATCTTCCAAGGTTACAGCTCGAGCTCTATAAGGAAATTAAAAAGAATGGTGCCCCTCGGAGTTTGCGTGCTGCCCTGTGGAGGTTTGCTGAGCTGGCTCACCTGGTTCGGCCTCAGAAATGCAGGCCTTACCTGGTGAACCTTCTGCCGTGCCTGACTCGAACAAGCAAGAGACCCGAAGAATCAGTCCAGGAGACCTTGGCTGCAGCTGTTCCCAAAATTATGGCTTCTTTTGGCAATTTTGCAAATGACAATGAAATTAAGGTTTTGTTAAAGGCCTTCATAGCGAACCTGAAGTCAAGCTCCCCCACCATTCGGCGGACAGCGGCTGGATCAGCAGTGAGCATCTGCCAGCACTCAAGAAGGACACAATATTTCTATAGTTGGCTACTAAATGTGCTCTTAGGCTTACTCGTTCCTGTCGAGGATGAACACTCCACTCTGCTGATTCTTGGCGTGCTGCTCACCCTGAGGTATTTGGTGCCCTTGCTGCAGCAGCAGGTCAAGGACACAAGCCTGAAAGGCAGCTTCGGAGTGACAAGGAAAGAAATGGAAGTCTCTCCTTCTGCAGAGCAGCTTGTCCAGGTTTATGAACTGACGTTACATCATACACAGCACCAAGACCACAATGTTGTGACCGGAGCCCTGGAGCTGTTGCAGCAGCTCTTCAGAACGCCTCCACCCGAGCTTCTGCAAACCCTGACCGCAGTCGGGGGCATTGGGCAGCTCACCGCTGCTAAGGAGGAGTCTGGTGGCCGAAGCCGTAGTGGGAGTATTGTGGAACTTATAGCTGGAGGGGGTTCCTCATGCAGCCCTGTCCTTTCAAGAAAACAAAAAGGCAAAGTGCTCTTAGGAGAAGAAGAAGCCTTGGAGGATGACTCTGAATCGAGATCGGATGTCAGCAGCTCTGCCTTAACAGCCTCAGTGAAGGATGAGATCAGTGGAGAGCTGGCTGCTTCTTCAGGGGTTTCCACTCCAGGGTCAGCAGGTCATGACATCATCACAGAACAGCCACGGTCACAGCACACACTGCAGGCGGACTCAGTGGATCTGGCCAGCTGTGACTTGACAAGCTCTGCCACTGATGGGGATGAGGAGGATATCTTGAGCCACAGCTCCAGCCAGGTCAGCGCCGTCCCATCTGACCCTGCCATGGACCTGTGAGGTACC |
| **Htt_1-550_Q89_(CAA/G)_ nucleotide sequence:**  AGATCTATGGCGACCCTGGAAAAGCTGATGAAGGCCTTCGAGTCCCTCAAGTCCTTCCAGCAACAGCAACAGCAACAGCAACAGCAACAGCAACAGCAACAGCAACAGCAACAGCAACAGCAACAGCAACAGCAACAGCAACAGCAACAGCAACAGCAACAGCAACAGCAACAGCAACAGCAACAGCAACAGCAACAGCAACAGCAACAGCAACAGCAACAGCAACAGCAACAGCAACAGCAACAGCAACAGCAACAGCAACAGCAACAGCAACAGCAACAGCAACAGCAACAGCAACAGCAACAGCAACAGCAACAGCAACAGCAACAGCCGCCACCGCCGCCGCCGCCGCCGCCGCCTCCTCAGCTTCCTCAGCCGCCGCCGCAGGCACAGCCGCTGCTGCCTCAGCCGCAGCCGCCCCCGCCGCCGCCCCCGCCGCCACCCGGCCCGGCTGTGGCTGAGGAGCCGCTGCACCGACCAAAGAAAGAACTTTCAGCTACCAAGAAAGACCGTGTGAATCATTGTCTGACAATATGTGAAAACATAGTGGCACAGTCTGTCAGAAATTCTCCAGAATTTCAGAAACTTCTGGGCATCGCTATGGAACTTTTTCTGCTGTGCAGTGATGACGCAGAGTCAGATGTCAGGATGGTGGCTGACGAATGCCTCAACAAAGTTATCAAAGCTTTGATGGATTCTAATCTTCCAAGGTTACAGCTCGAGCTCTATAAGGAAATTAAAAAGAATGGTGCCCCTCGGAGTTTGCGTGCTGCCCTGTGGAGGTTTGCTGAGCTGGCTCACCTGGTTCGGCCTCAGAAATGCAGGCCTTACCTGGTGAACCTTCTGCCGTGCCTGACTCGAACAAGCAAGAGACCCGAAGAATCAGTCCAGGAGACCTTGGCTGCAGCTGTTCCCAAAATTATGGCTTCTTTTGGCAATTTTGCAAATGACAATGAAATTAAGGTTTTGTTAAAGGCCTTCATAGCGAACCTGAAGTCAAGCTCCCCCACCATTCGGCGGACAGCGGCTGGATCAGCAGTGAGCATCTGCCAGCACTCAAGAAGGACACAATATTTCTATAGTTGGCTACTAAATGTGCTCTTAGGCTTACTCGTTCCTGTCGAGGATGAACACTCCACTCTGCTGATTCTTGGCGTGCTGCTCACCCTGAGGTATTTGGTGCCCTTGCTGCAGCAGCAGGTCAAGGACACAAGCCTGAAAGGCAGCTTCGGAGTGACAAGGAAAGAAATGGAAGTCTCTCCTTCTGCAGAGCAGCTTGTCCAGGTTTATGAACTGACGTTACATCATACACAGCACCAAGACCACAATGTTGTGACCGGAGCCCTGGAGCTGTTGCAGCAGCTCTTCAGAACGCCTCCACCCGAGCTTCTGCAAACCCTGACCGCAGTCGGGGGCATTGGGCAGCTCACCGCTGCTAAGGAGGAGTCTGGTGGCCGAAGCCGTAGTGGGAGTATTGTGGAACTTATAGCTGGAGGGGGTTCCTCATGCAGCCCTGTCCTTTCAAGAAAACAAAAAGGCAAAGTGCTCTTAGGAGAAGAAGAAGCCTTGGAGGATGACTCTGAATCGAGATCGGATGTCAGCAGCTCTGCCTTAACAGCCTCAGTGAAGGATGAGATCAGTGGAGAGCTGGCTGCTTCTTCAGGGGTTTCCACTCCAGGGTCAGCAGGTCATGACATCATCACAGAACAGCCACGGTCACAGCACACACTGCAGGCGGACTCAGTGGATCTGGCCAGCTGTGACTTGACAAGCTCTGCCACTGATGGGGATGAGGAGGATATCTTGAGCCACAGCTCCAGCCAGGTCAGCGCCGTCCCATCTGACCCTGCCATGGACCTGTGAGGTACC |

**Supplementary Table 2. Demographic information of the control and HD iPSCs used in this study**

| **Control/Patient** | **Line ID** | **CAG repeat number**  **(expanded allele)** | **Age** | **Gender** |
| --- | --- | --- | --- | --- |
| Healthy control_a | SFC-840-03-03 | - | 36 – 78 | Female |
| Healthy control_b | SFC-841-03-01 | - |  | Male |
| Healthy control_c | SFC-856-03-04 | - |  | Female |
| HD_a | ND36998 | 60 | 20 – 29 | Female |
| HD_b | ND41656 | 57 |  | Female |
| HD_c | ND42230 | 71 |  | Female |

**Supplementary Table 3. Demographic information of the control and HD patients**

| **Unaffected/HD** | **Case No.** | **HD grade** | **CAG repeat lengths** | **Age-at-death (years)** | **Gender** | **Post-mortem delay (hours)** |
| --- | --- | --- | --- | --- | --- | --- |
| Unaffected_1 | H170 | - | 10/17 | 22 – 78 | Male | 17 |
| Unaffected_2 | H174 | - | 17/18 |  | Male | 24.5 |
| Unaffected_3 | H181 | - | 18/19 |  | Female | 20 |
| Unaffected_4 | H194 | - | 17/19 |  | Male | 22.5 |
| Unaffected_5 | H209 | - | 17/20 |  | Male | 23 |
| Unaffected_6 | H239 | - | 13/15 |  | Male | 15.5 |
| Unaffected_7 | H177 | - | 11/23 |  | Male | 21 |
| HD_1 | HC132 | HD-1 | 17/47 | 32 – 70 | Male | 14 |
| HD_2 | HC133 | HD-2 | 17/43 |  | Male | 14 |
| HD_3 | HC150 | HD-2 | 22/42 |  | Female | 21 |
| HD_4 | HC161 | HD-2 | 17/42 |  | Female | 8 |
| HD_5 | HC147 | HD-3 | 27/42 |  | Male | 18 |
| HD_6 | HC148 | HD-3 | 22/43 |  | Male | 16 |
| HD_7 | HC77 | HD-4 | 17/54 |  | Female | 9 |

**Supplementary Table 4. List of primers used in this study**

| **qPCR primers** | |
| --- | --- |
| **Primer names** | **Sequences (5’ to 3’)** |
| Human *PAPD5*-forward | TGCCCCTAGAGACGACCAA |
| Human *PAPD5*-reverse | GTAGTTGAGTCCATACGTGCTG |
| Human *Htt1-550*-forward | ACCCGAAGAATCAGTCCAGG |
| Human *Htt1-550*-reverse | GAGCTTGACTTCAGGTTCGC |
| Human *miR-7-5p*-forward | CGCAGTGGAAGACTAGTGA |
| Human *miR-7-5p*-reverse | CGAATTCTAGAGCTCGAGG |
| Human *RNU66*-forward | GTAACTGTGGTGATGGAAATGTG |
| Human *RNU66*-reverse | GACTGTACTAGGATAGAAAGAACC |
| Fly *dPAPD5*-forward | TAGACTACTACGGCCGCAAG |
| Fly *dPAPD5*-reverse | AGCTCCTCCCTATGTCGTTG |
| Fly *dYY1*-forward | TACATTCGAGGGATGCGGAA |
| Fly *dYY1*-reverse | TGAACAACCACTTTTCCCCG |
| *β-actin*/*dβ-actin*-forward | ATGTGCAAGGCCGGTTTCGC |
| *β-actin*/*dβ-actin*-reverse | CGACACGCAGCTCATTGTAG |

**Supplementary Table 5. Summary of the fly genotypes in this study**

| **Fig. 1h** | *w*; *GMR-GAL4 UAS*-*DsRed_CAG0_*/+; +/+ |
| --- | --- |
|  | *w*; *GMR-GAL4 UAS*-*DsRed_CAG0_*/+; *UAS-dPAPD5-dsRNA^GD19799^*/+ |
|  | *w*; *GMR-GAL4*/+; *UAS*-*DsRed_CAG100_*/+ |
|  | *w*; *GMR-GAL4*/+; *UAS*-*DsRed_CAG100_*/*UAS-dPAPD5-dsRNA^GD19799^* |
| **Fig. 4b** | *w*; *GMR-GAL4*/+; +/+ |
|  | *w*; *GMR-GAL4*/+; *UAS-dPAPD5-dsRNA^GD19799^*/+ |
|  | *w*; *GMR-GAL4*/+; *UAS*-*Htt_exon1_Q93*/+ |
|  | *w*; *GMR-GAL4*/+; *UAS*-*Htt_exon1_Q93/UAS-dPAPD5-dsRNA^GD19799^* |
| **Fig. 4e** | *w*; *Tub56D^Gene-Switch^-GAL4*/+; +/+ |
|  | *w*; *Tub56D^Gene-Switch^-GAL4*/+; *UAS-dPAPD5-dsRNA^GD19799^*/+ |
|  | *w*; *Tub56D^Gene-Switch^-GAL4*/+; *UAS*-*Htt_exon1_Q93*/+ |
|  | *w*; *Tub56D^Gene-Switch^-GAL4*/+; *UAS*-*Htt_exon1_Q93/UAS-dPAPD5-dsRNA^GD19799^* |
| **Fig. 7b** | *w*; *GMR-GAL4 UAS*-*DsRed_CAG0_*/+; +/+ |
|  | *w*; *GMR-GAL4 UAS*-*DsRed_CAG0_*/+; *UAS-dYY1-dsRNA^GD39529^*/+ |
|  | *w*; *GMR-GAL4*/+; *UAS*-*DsRed_CAG100_*/+ |
|  | *w*; *GMR-GAL4*/+; *UAS*-*DsRed_CAG100_*/*UAS-dYY1-dsRNA^GD39529^* |
| **Fig. 7e** | *w*; *GMR-GAL4*/+; +/+ |
|  | *w*; *GMR-GAL4*/+; *UAS-dYY1-dsRNA^GD39529^*/+ |
|  | *w*; *GMR-GAL4*/+; *UAS*-*Htt_exon1_Q93*/+ |
|  | *w*; *GMR-GAL4*/+; *UAS*-*Htt_exon1_Q93/UAS-dYY1-dsRNA^GD39529^* |
| **Fig. 7h** | *w*; *GMR-GAL4 UAS*-*DsRed_CAG0_*/+; +/+ |
|  | *w*; *GMR-GAL4 UAS*-*DsRed_CAG0_*/+; *UAS-dYY1*/+ |
|  | *w*; *GMR-GAL4*/+; *UAS*-*DsRed_CAG100_*/+ |
|  | *w*; *GMR-GAL4*/+; *UAS*-*DsRed_CAG100_*/*UAS-dYY1* |
| **Fig. 7k** | *w*; *GMR-GAL4*/+; +/+ |
|  | *w*; *GMR-GAL4*/+; *UAS-dYY1*/+ |
|  | *w*; *GMR-GAL4*/+; *UAS*-*Htt_exon1_Q93*/+ |
|  | *w*; *GMR-GAL4*/+; *UAS*-*Htt_exon1_Q93/UAS-dYY1* |
| **Fig. 7m** | *w*; *GMR-GAL4*/+; *UAS*-*Htt_exon1_Q93*/+ |
|  | *w*; *GMR-GAL4*/+; *UAS*-*Htt_exon1_Q93*/*UAS-dYY1* |
|  | *w*; *GMR-GAL4*/+; *UAS*-*Htt_exon1_Q93*/*UAS-dPAPD5-dsRNA^GD19799^* |
|  | *w*; *GMR-GAL4*/+; *UAS*-*Htt_exon1_Q93*/*UAS-dYY1* *UAS-dPAPD5-dsRNA^GD19799^* |
| **Supplementary Fig. 1** | *w*; *GMR-GAL4*/+; +/+ |
|  | *w*; *GMR-GAL4*/+; *UAS-dPAPD5-dsRNA^GD19799^*/+ |
|  | *w*; *GMR-GAL4* *UAS*-*CGG_90_-EGFP*/+; +/+ |
|  | *w*; *GMR-GAL4* *UAS-CGG_90_*-*EGFP*/+; *UAS-dPAPD5-dsRNA^GD19799^*/+ |
|  | *w*; *GMR-GAL4*/+; *UAS*-*CTG_60_*/+ |
|  | *w*; *GMR-GAL4*/+; *UAS*-*CTG_60_*/*UAS-dPAPD5-dsRNA^GD19799^* |
|  | *w*; *GMR-GAL4*/+; *UAS*-*CTG_480_*/+ |
|  | *w*; *GMR-GAL4*/+; *UAS*-*CTG_480_*/*UAS-dPAPD5-dsRNA^GD19799^* |

**Supplementary Table 6. Summary of the exact *P* values in main and supplementary figures.**

| **Fig. 1a** Statistical analysis was performed using one-way ANOVA followed by *post hoc* Tukey's test: Untransfected vs EGFPCAG27, *P* = 0.8988; Untransfected vs EGFPCAG78, **P* = 0.0208; EGFPCAG27 vs EGFPCAG78, **P* = 0.0349.  **Fig. 1c** Statistical analysis was performed using one-way ANOVA followed by *post hoc* Tukey's test: Untransfected vs EGFPCAG27, *P* = 0.8184; Untransfected vs EGFPCAG78, ****P* = 0.0002; EGFPCAG27 vs EGFPCAG78, ****P* = 0.0003.  **Fig. 1e** Statistical analysis was performed using two-tailed unpaired Student's *t*-test: Control-siRNA vs PAPD5-siRNA, **P* = 0.0149.  **Fig. 1f** Statistical analysis was performed using one-way ANOVA followed by *post hoc* Tukey's test: Untransfected + Control-siRNA vs Untransfected + PAPD5-siRNA, *P* = 0.9992; EGFPCAG27 + Control-siRNA vs EGFPCAG27 + PAPD5-siRNA, *P* = 0.9802; EGFPCAG78 + Control-siRNA vs EGFPCAG78 + PAPD5-siRNA, ***P* = 0.0021; Untransfected + Control-siRNA vs EGFPCAG27 + Control-siRNA, *P* > 0.9999; EGFPCAG27 + Control-siRNA vs EGFPCAG78 + Control-siRNA, ****P* = 0.0006.  **Fig. 1g** Statistical analysis was performed using one-way ANOVA followed by *post hoc* Tukey's test: CAG0 vs CAG0 + dPAPD5-dsRNA, **P* = 0.0485; CAG0 vs CAG100, **P* = 0.0404; CAG100 vs CAG100 + dPAPD5-dsRNA, ***P* = 0.0015.  **Fig. 1i** Statistical analysis was performed using one-way ANOVA followed by *post hoc* Tukey's test: CAG0 vs CAG0 + dPAPD5-dsRNA, *P* = 0.6781; CAG0 vs CAG100, *****P* < 0.0001; CAG100 vs CAG100 + dPAPD5-dsRNA, *****P* < 0.0001.  **Fig. 1k** Statistical analysis was performed using one-way ANOVA followed by *post hoc* Tukey's test:  1) **PAPD5**: Untransfected vs Empty vector, *P* = 0.9928; Untransfected vs PAPD5 (0.5 μg), *P* = 0.9975; Untransfected vs PAPD5 (1.0 μg), *****P* < 0.0001;  2) **PAPD5^D256A & D258A^**: Untransfected vs Empty vector, *P* = 0.9616; Untransfected vs PAPD5 (0.5 μg), *P* = 0.8061; Untransfected vs PAPD5 (1.0 μg), *P* = 0.3715.  **Fig. 1l** Statistical analysis was performed using one-way ANOVA followed by *post hoc* Tukey's test:  1) **PAPD5**: Untransfected vs Empty vector, *P* = 0.9743; Untransfected vs PAPD5 (0.5 μg), *P* = 0.4941; Untransfected vs PAPD5 (1.0 μg), ***P* = 0.0094;  2) **PAPD5^D256A & D258A^**: Untransfected vs Empty vector, *P* = 0.9837; Untransfected vs PAPD5 (0.5 μg), *P* = 0.3862; Untransfected vs PAPD5 (1.0 μg), *P* = 0.0737.  **Fig. 1o, r** Statistical analysis was performed using one-way ANOVA followed by *post hoc* Tukey's test:  1) **p-MKK4**: Untransfected vs Empty vector, *P* = 0.7038; Untransfected vs PAPD5 (0.5 μg), *P* = 0.1032; Untransfected vs PAPD5 (1.0 μg), ***P* = 0.0028;  2) **p-JNK**: Untransfected vs Empty vector, *P* = 0.4857; Untransfected vs PAPD5 (0.5 μg), *P* = 0.1642; Untransfected vs PAPD5 (1.0 μg), **P* = 0.0249;  3) **p-TAK1**: Untransfected vs Empty vector, *P* = 0.7084; Untransfected vs PAPD5 (0.5 μg), *P* = 0.6149; Untransfected vs PAPD5 (1.0 μg), **P* = 0.0266.  **Fig. 1t** Statistical analysis was performed using one-way ANOVA followed by *post hoc* Tukey's test: Untransfected vs Untransfected + JNK inhibitor, *P* = 0.9967; Untransfected vs PAPD5, *****P* < 0.0001; PAPD5 vs PAPD5 + JNK inhibitor (0.5 μM), ****P* = 0.0001; PAPD5 vs PAPD5 + JNK inhibitor (1.0 μM), *****P* < 0.0001; PAPD5 vs PAPD5 + JNK inhibitor (2.0 μM), *****P* < 0.0001.  **Fig. 1u** Statistical analysis was performed using one-way ANOVA followed by *post hoc* Tukey's test: Untransfected vs Untransfected + JNK inhibitor, *P* = 0.9984; Untransfected vs PAPD5, *****P* < 0.0001; PAPD5 vs PAPD5 + JNK inhibitor (0.5 μM), *P* = 0.2684; PAPD5 vs PAPD5 + JNK inhibitor (1.0 μM), ***P* = 0.0025; PAPD5 vs PAPD5 + JNK inhibitor (2.0 μM), *****P* < 0.0001.  **Fig. 1w** Statistical analysis was performed using one-way ANOVA followed by *post hoc* Tukey's test:  1) **p-MKK4**: Untransfected vs Untransfected + TAK1 inhibitor, *P* = 0.9993; Untransfected vs PAPD5, ****P* = 0.0002; PAPD5 vs PAPD5 + TAK1 inhibitor (0.5 μM), *P* = 0.2198; PAPD5 vs PAPD5 + TAK1 inhibitor (1.0 μM), *P* = 0.1346; PAPD5 vs PAPD5 + TAK1 inhibitor (2.0 μM), ***P* = 0.0037;  2) **p-JNK**: Untransfected vs Untransfected + TAK1 inhibitor, *P* > 0.9999; Untransfected vs PAPD5, ***P* = 0.0011; PAPD5 vs PAPD5 + TAK1 inhibitor (0.5 μM), **P* = 0.0231; PAPD5 vs PAPD5 + TAK1 inhibitor (1.0 μM), ***P* = 0.0019; PAPD5 vs PAPD5 + TAK1 inhibitor (2.0 μM), ****P* = 0.0009;  3) **cleaved caspase-3**: Untransfected vs Untransfected + TAK1 inhibitor, *P* > 0.9999; Untransfected vs PAPD5, **P* = 0.0104; PAPD5 vs PAPD5 + TAK1 inhibitor (0.5 μM), *P* = 0.0852; PAPD5 vs PAPD5 + TAK1 inhibitor (1.0 μM), **P* = 0.0486; PAPD5 vs PAPD5 + TAK1 inhibitor (2.0 μM), **P* = 0.0146.  **Fig. 1x** Statistical analysis was performed using one-way ANOVA followed by *post hoc* Tukey's test: Untransfected vs Untransfected + TAK1 inhibitor, *P* = 0.9904; Untransfected vs PAPD5, **P* = 0.0140; PAPD5 vs PAPD5 + TAK1 inhibitor (0.5 μM), *P* = 0.5169; PAPD5 vs PAPD5 + TAK1 inhibitor (1.0 μM), **P* = 0.0400; PAPD5 vs PAPD5 + TAK1 inhibitor (2.0 μM), **P* = 0.0206. |
| --- |
| **Fig. 2b** Statistical analysis was performed using one-way ANOVA followed by *post hoc* Tukey's test:  1) **p-TAK1**: Untransfected + Control-siRNA vs Untransfected + PAPD5-siRNA, *P* = 0.9865; EGFPCAG27 + Control-siRNA vs EGFPCAG27 + PAPD5-siRNA, *P* = 0.9998; EGFPCAG78 + Control-siRNA vs EGFPCAG78 + PAPD5-siRNA, **P* = 0.0347; Untransfected + Control-siRNA vs EGFPCAG27 + Control-siRNA, *P* = 0.9759; EGFPCAG27 + Control-siRNA vs EGFPCAG78 + Control-siRNA, **P* = 0.0162;  2) **p-MKK4**: Untransfected + Control-siRNA vs Untransfected + PAPD5-siRNA, *P* = 0.9979; EGFPCAG27 + Control-siRNA vs EGFPCAG27 + PAPD5-siRNA, *P* = 0.9990; EGFPCAG78 + Control-siRNA vs EGFPCAG78 + PAPD5-siRNA, ***P* = 0.0018; Untransfected + Control-siRNA vs EGFPCAG27 + Control-siRNA, *P* = 0.8699; EGFPCAG27 + Control-siRNA vs EGFPCAG78 + Control-siRNA, ***P* = 0.0018;  3) **p-JNK**: Untransfected + Control-siRNA vs Untransfected + PAPD5-siRNA, *P* = 0.9988; EGFPCAG27 + Control-siRNA vs EGFPCAG27 + PAPD5-siRNA, *P* = 0.9998; EGFPCAG78 + Control-siRNA vs EGFPCAG78 + PAPD5-siRNA, *****P* < 0.0001; Untransfected + Control-siRNA vs EGFPCAG27 + Control-siRNA, *P* > 0.9999; EGFPCAG27 + Control-siRNA vs EGFPCAG78 + Control-siRNA, *****P* < 0.0001;  4) **cleaved caspase-3**: Untransfected + Control-siRNA vs Untransfected + PAPD5-siRNA, *P* > 0.9999; EGFPCAG27 + Control-siRNA vs EGFPCAG27 + PAPD5-siRNA, *P* = 0.9948; EGFPCAG78 + Control-siRNA vs EGFPCAG78 + PAPD5-siRNA, **P* = 0.0207; Untransfected + Control-siRNA vs EGFPCAG27 + Control-siRNA, *P* = 0.9884; EGFPCAG27 + Control-siRNA vs EGFPCAG78 + Control-siRNA, ***P* = 0.0011;  Statistical analysis was performed using two-tailed unpaired Student's *t*-test:  5) **PAPD5**: Statistical analysis was performed using two-tailed unpaired Student's *t*-test: Untransfected + Control-siRNA vs Untransfected + PAPD5-siRNA, ***P* = 0.0018; EGFPCAG27 + Control-siRNA vs EGFPCAG27 + PAPD5-siRNA, ***P* = 0.0089; EGFPCAG78 + Control-siRNA vs EGFPCAG78 + PAPD5-siRNA, *P* = 0.0523.  **Fig. 2c** Statistical analysis was performed using one-way ANOVA followed by *post hoc* Tukey's test: Untransfected vs Untransfected + TAK1 inhibitor, *P* = 0.9994; Untransfected vs EGFPCAG78, ***P* = 0.0058; EGFPCAG78 vs EGFPCAG78 + TAK1 inhibitor (0.5 μM), *P* = 0.7227; EGFPCAG78 vs EGFPCAG78 + TAK1 inhibitor (1.0 μM), *P* = 0.2426; EGFPCAG78 vs EGFPCAG78 + TAK1 inhibitor (2.0 μM), ***P* = 0.0090.  **Fig. 2e** Statistical analysis was performed using one-way ANOVA followed by *post hoc* Tukey's test:  1) **p-MKK4**: Untransfected vs Untransfected + TAK1 inhibitor, *P* > 0.9999; Untransfected vs EGFPCAG78, *****P* < 0.0001; EGFPCAG78 vs EGFPCAG78 + TAK1 inhibitor (0.5 μM), ***P* = 0.0014; EGFPCAG78 vs EGFPCAG78 + TAK1 inhibitor (1.0 μM), *****P* < 0.0001; EGFPCAG78 vs EGFPCAG78 + TAK1 inhibitor (2.0 μM), *****P* < 0.0001;  2) **p-JNK**: Untransfected vs Untransfected + TAK1 inhibitor, *P* = 0.9999; Untransfected vs EGFPCAG78, *****P* < 0.0001; EGFPCAG78 vs EGFPCAG78 + TAK1 inhibitor (0.5 μM), ***P* = 0.0060; EGFPCAG78 vs EGFPCAG78 + TAK1 inhibitor (1.0 μM), ****P* = 0.0006; EGFPCAG78 vs EGFPCAG78 + TAK1 inhibitor (2.0 μM), ****P* = 0.0006;  3) **cleaved caspase-3**: Untransfected vs Untransfected + TAK1 inhibitor, *P* > 0.9999; Untransfected vs EGFPCAG78, ****P* = 0.0001; EGFPCAG78 vs EGFPCAG78 + TAK1 inhibitor (0.5 μM), **P* = 0.0325; EGFPCAG78 vs EGFPCAG78 + TAK1 inhibitor (1.0 μM), ***P* = 0.0018; EGFPCAG78 vs EGFPCAG78 + TAK1 inhibitor (2.0 μM), ****P* = 0.0002.  **Fig. 2f** Statistical analysis was performed using one-way ANOVA followed by *post hoc* Tukey's test: Untransfected vs Untransfected + JNK inhibitor, *P* > 0.9999; Untransfected vs EGFPCAG78, ****P* = 0.0005; EGFPCAG78 vs EGFPCAG78 + JNK inhibitor (0.5 μM), *P* = 0.7299; EGFPCAG78 vs EGFPCAG78 + JNK inhibitor (1.0 μM), *P* = 0.0788; EGFPCAG78 vs EGFPCAG78 + JNK inhibitor (2.0 μM), ***P* = 0.0014.  **Fig. 2h** Statistical analysis was performed using one-way ANOVA followed by *post hoc* Tukey's test: Untransfected vs Untransfected + JNK inhibitor, *P* > 0.9999; Untransfected vs EGFPCAG78, ***P* = 0.0053; EGFPCAG78 vs EGFPCAG78 + JNK inhibitor (0.5 μM), *P* = 0.0629; EGFPCAG78 vs EGFPCAG78 + JNK inhibitor (1.0 μM), **P* = 0.0181; EGFPCAG78 vs EGFPCAG78 + JNK inhibitor (2.0 μM), ***P* = 0.0057. |
| **Fig. 3e** Statistical analysis was performed using one-way ANOVA followed by *post hoc* Tukey's test:  1) **PAPD5**: Untransfected vs Empty vector, *P* > 0.9999; Untransfected vs PAPD5 (0.5 μg), *P* = 0.9859; Untransfected vs PAPD5 (1.0 μg), **P* = 0.0181;  2) **PAPD5^D256A & D258A^**: Untransfected vs Empty vector, *P* > 0.9999; Untransfected vs PAPD5 (0.5 μg), *P* = 0.9957; Untransfected vs PAPD5 (1.0 μg), *P* = 0.9233.  **Fig. 3f** Statistical analysis was performed using one-way ANOVA followed by *post hoc* Fisher's LSD test: Untransfected vs PAPD5, **P* = 0.0263; PAPD5 vs PAPD5^D256A & D258A^, **P* = 0.0251, Untransfected vs PAPD5^D256A & D258A^, *P* = 0.9732.  **Fig. 3g** Statistical analysis was performed using two-tailed unpaired Student's *t*-test: Control-siRNA vs PAPD5-siRNA, **P* = 0.0118.  **Fig. 3h** Statistical analysis was performed using one-way ANOVA followed by *post hoc* Tukey's test: Untransfected vs Untransfected + miR-7, *P* = 0.9514; Untransfected vs EGFPCAG78, *****P* < 0.0001; EGFPCAG78 vs EGFPCAG78 + miR-7 (0.25 μg), *****P* < 0.0001; EGFPCAG78 vs EGFPCAG78 + miR-7 (0.5 μg), *****P* < 0.0001; EGFPCAG78 vs EGFPCAG78 + miR-7 (1.0 μg), *****P* < 0.0001.  **Fig. 3j** Statistical analysis was performed using one-way ANOVA followed by *post hoc* Tukey's test:  1) **TAB2**: Untransfected vs Untransfected + miR-7, *P* > 0.9999; Untransfected vs EGFPCAG78, ***P* = 0.0085; EGFPCAG78 vs EGFPCAG78 + miR-7 (0.25 μg), *P* = 0.8775; EGFPCAG78 vs EGFPCAG78 + miR-7 (0.5 μg), *P* = 0.1140; EGFPCAG78 vs EGFPCAG78 + miR-7 (1.0 μg), **P* = 0.0463;  2) **p-TAK1**: Untransfected vs Untransfected + miR-7, *P* = 0.9975; Untransfected vs EGFPCAG78, ***P* = 0.0050; EGFPCAG78 vs EGFPCAG78 + miR-7 (0.25 μg), *P* = 0.6145; EGFPCAG78 vs EGFPCAG78 + miR-7 (0.5 μg), *P* = 0.0531; EGFPCAG78 vs EGFPCAG78 + miR-7 (1.0 μg), ***P* = 0.0054;  3) **p-JNK**: Untransfected vs Untransfected + miR-7, *P* > 0.9999; Untransfected vs EGFPCAG78, ***P* = 0.0075; EGFPCAG78 vs EGFPCAG78 + miR-7 (0.25 μg), *P* = 0.7101; EGFPCAG78 vs EGFPCAG78 + miR-7 (0.5 μg), *P* = 0.0832; EGFPCAG78 vs EGFPCAG78 + miR-7 (1.0 μg), **P* = 0.0179;  4) **cleaved caspase-3**: Untransfected vs Untransfected + miR-7, *P* > 0.9999; Untransfected vs EGFPCAG78, ***P* = 0.0027; EGFPCAG78 vs EGFPCAG78 + miR-7 (0.25 μg), *P* = 0.3808; EGFPCAG78 vs EGFPCAG78 + miR-7 (0.5 μg), *P* = 0.0775; EGFPCAG78 vs EGFPCAG78 + miR-7 (1.0 μg), ***P* = 0.0049. |
| **Fig. 4a** Statistical analysis was performed using one-way ANOVA followed by *post hoc* Tukey's test: GMR vs GMR + dPAPD5-dsRNA, *P* = 0.0864; GMR vs GMR > Q93, **P* = 0.0478; GMR > Q93 vs GMR > Q93 + dPAPD5-dsRNA, ***P* = 0.0016.  **Fig. 4c** Statistical analysis was performed using one-way ANOVA followed by *post hoc* Tukey's test: GMR vs GMR + dPAPD5-dsRNA, *P* = 0.8006; GMR vs GMR > HttQ93, *****P* < 0.0001; GMR > HttQ93 vs GMR > HttQ93 + dPAPD5-dsRNA, *****P* < 0.0001.  **Fig. 4d** Statistical analysis was performed using one-way ANOVA followed by *post hoc* Tukey's test: Tub vs Tub + dPAPD5-dsRNA, *****P* < 0.0001; Tub vs Tub > Q93, *****P* < 0.0001; Tub > Q93 vs Tub > Q93 + dPAPD5-dsRNA, *****P* < 0.0001.  **Fig. 4e** Statistical analysis was performed using one-way ANOVA followed by *post hoc* Tukey's test: Tub56D^Gene-Switch^ vs Tub56D^Gene-Switch^ + dPAPD5-dsRNA, *P* = 0.9656; Tub56D^Gene-Switch^ vs Tub56D^Gene-Switch^ > HttQ93, *****P* < 0.0001; Tub56D^Gene-Switch^ > HttQ93 vs Tub56D^Gene-Switch^ > HttQ93 + dPAPD5-dsRNA, *****P* < 0.0001.  **Fig. 4f** Statistical analysis was performed using one-way ANOVA followed by *post hoc* Tukey's test: Untransfected vs HttQ23, *P* = 0.9832; Untransfected vs HttQ89, **P* = 0.0253; HttQ23 vs HttQ89, **P* = 0.0207.  **Fig. 4h** Statistical analysis was performed using one-way ANOVA followed by *post hoc* Tukey's test: Untransfected vs HttQ23, *P* = 0.9920; Untransfected vs HttQ89, *****P* < 0.0001; HttQ23 vs HttQ89, *****P* < 0.0001.  **Fig. 4i** Statistical analysis was performed using two-tailed unpaired Student's *t*-test: Control-siRNA vs Huntingtin-siRNA, *P* = 0.4859.  **Fig. 4k** Statistical analysis was performed using two-tailed unpaired Student's *t*-test:  1) **PAPD5**: Control-siRNA vs Huntingtin-siRNA, *P* = 0.4465;  2) **Huntingtin**: Control-siRNA vs Huntingtin-siRNA, **P* = 0.0305.  **Fig. 4l** Statistical analysis was performed using one-way ANOVA followed by *post hoc* Tukey's test: Untransfected + Control-siRNA vs Untransfected + PAPD5-siRNA, *P* = 0.9755; HttQ23 + Control-siRNA vs HttQ23 + PAPD5-siRNA, *P* = 0.9971; HttQ89 + Control-siRNA vs HttQ89 + PAPD5-siRNA, *****P* < 0.0001; Untransfected + Control-siRNA vs HttQ23 + Control-siRNA, **P* = 0.0378; HttQ23 + Control-siRNA vs HttQ89 + Control-siRNA, *****P* < 0.0001.  **Fig. 4n** Statistical analysis was performed using one-way ANOVA followed by *post hoc* Tukey's test:  1) **p-TAK1**: Untransfected + Control-siRNA vs Untransfected + PAPD5-siRNA, *P* = 0.9997; Q23 + Control-siRNA vs HttQ23 + PAPD5-siRNA, *P* = 0.9986; HttQ89 + Control-siRNA vs HttQ89 + PAPD5-siRNA, **P* = 0.0222; Untransfected + Control-siRNA vs HttQ23 + Control-siRNA, *P* = 0.9997; HttQ23 + Control-siRNA vs HttQ89 + Control-siRNA, ****P* = 0.0001;  2) **p-MKK4**: Untransfected + Control-siRNA vs Untransfected + PAPD5-siRNA, *P* = 0.9835; HttQ23 + Control-siRNA vs HttQ23 + PAPD5-siRNA, *P* > 0.9999; HttQ89 + Control-siRNA vs HttQ89 + PAPD5-siRNA, *****P* < 0.0001; Untransfected + Control-siRNA vs HttQ23 + Control-siRNA, *P* = 0.9558; HttQ23 + Control-siRNA vs HttQ89 + Control-siRNA, *****P* < 0.0001;  3) **p-JNK**: Untransfected + Control-siRNA vs Untransfected + PAPD5-siRNA, *P* > 0.9999; HttQ23 + Control-siRNA vs HttQ23 + PAPD5-siRNA, *P* = 0.9996; HttQ89 + Control-siRNA vs HttQ89 + PAPD5-siRNA, ****P* = 0.0003; Untransfected + Control-siRNA vs HttQ23 + Control-siRNA, *P* > 0.9999; HttQ23 + Control-siRNA vs HttQ89 + Control-siRNA, *****P* < 0.0001;  4) **cleaved caspase-3**: Untransfected + Control-siRNA vs Untransfected + PAPD5-siRNA, *P* = 0.9999; HttQ23 + Control-siRNA vs HttQ23 + PAPD5-siRNA, *P* = 0.9998; HttQ89 + Control-siRNA vs HttQ89 + PAPD5-siRNA, ***P* = 0.0011; Untransfected + Control-siRNA vs HttQ23 + Control-siRNA, *P* = 0.9652; HttQ23 + Control-siRNA vs HttQ89 + Control-siRNA, *****P* < 0.0001;  5) **PAPD5**: Statistical analysis was performed using two-tailed unpaired Student's *t*-test: Untransfected + Control-siRNA vs Untransfected + PAPD5-siRNA, **P* = 0.0287; HttQ23 + Control-siRNA vs HttQ23 + PAPD5-siRNA, **P* = 0.0424; HttQ89 + Control-siRNA vs HttQ89 + PAPD5-siRNA, ***P* = 0.0015.  **Fig. 4p** Statistical analysis was performed using one-way ANOVA followed by *post hoc* Tukey's test: Untransfected vs Untransfected + JNK inhibitor, *P* > 0.9999; Untransfected vs HttQ23, *P* = 0.9335; HttQ23 vs HttQ23 + JNK inhibitor, *P* > 0.9999; HttQ23 vs HttQ89, *****P* < 0.0001; HttQ89 vs HttQ89 + JNK inhibitor, *****P* < 0.0001.  **Fig. 4q** Statistical analysis was performed using one-way ANOVA followed by *post hoc* Tukey's test: Untransfected vs Untransfected + JNK inhibitor, *P* > 0.9999; Untransfected vs HttQ23, *P* = 0.9989; HttQ23 vs HttQ23 + JNK inhibitor, *P* = 0.9994; HttQ23 vs HttQ89, **P* = 0.0237; HttQ89 vs HttQ89 + JNK inhibitor, **P* = 0.0289.  **Fig. 4s** Statistical analysis was performed using one-way ANOVA followed by *post hoc* Tukey's test:  1) **p-MKK4**: Untransfected vs Untransfected + TAK1 inhibitor, *P* = 0.9994; HttQ23 vs HttQ23 + TAK1 inhibitor, *P* = 0.9987; HttQ89 vs HttQ89 + TAK1 inhibitor, ***P* = 0.0080; Untransfected vs HttQ23, *P* = 0.9994; HttQ23 vs HttQ89, ****P* = 0.0003;  2) **p-JNK**: Untransfected vs Untransfected + TAK1 inhibitor, *P* = 0.9945; HttQ23 vs HttQ23 + TAK1 inhibitor, *P* = 0.9976; HttQ89 vs HttQ89 + TAK1 inhibitor, *****P* < 0.0001; Untransfected vs HttQ23, *P* > 0.9999; HttQ23 vs HttQ89, *****P* < 0.0001;  3) **cleaved caspase-3**: Untransfected vs Untransfected + TAK1 inhibitor, *P* = 0.9982; HttQ23 vs HttQ23 + TAK1 inhibitor, *P* = 0.9997; HttQ89 vs HttQ89 + TAK1 inhibitor, *****P* < 0.0001; Untransfected vs HttQ23, *P* = 0.9999; HttQ23 vs HttQ89, *****P* < 0.0001.  **Fig. 4t** Statistical analysis was performed using one-way ANOVA followed by *post hoc* Tukey's test: Untransfected vs Untransfected + TAK1 inhibitor, *P* = 0.9570; Untransfected vs HttQ23, *P* = 0.9972; HttQ23 vs HttQ23 + TAK1 inhibitor, *P* = 0.9984; HttQ23 vs HttQ89, ***P* = 0.0014; HttQ89 vs HttQ89 + TAK1 inhibitor, ***P* = 0.0032.  **Fig. 4v** Statistical analysis was performed using one-way ANOVA followed by *post hoc* Tukey's test:  1) **TAB2**: Untransfected vs Untransfected + miR-7, *P* = 0.9964; Untransfected vs HttQ89, **P* = 0.0463; HttQ89 vs HttQ89 + miR-7 (0.25 μg), *P* = 0.2142; HttQ89 vs HttQ89 + miR-7 (0.5 μg), *P* = 0.2486; HttQ89 vs HttQ89 + miR-7 (1.0 μg), **P* = 0.0498;  2) **p-TAK1**: Untransfected vs Untransfected + miR-7, *P* = 0.9997; Untransfected vs HttQ89, **P* = 0.0220; HttQ89 vs HttQ89 + miR-7 (0.25 μg), *P* = 0.2497; HttQ89 vs HttQ89 + miR-7 (0.5 μg), *P* = 0.0864; HttQ89 vs HttQ89 + miR-7 (1.0 μg), **P* = 0.0217;  3) **p-MKK4**: Untransfected vs Untransfected + miR-7, *P* > 0.9999; Untransfected vs HttQ89, *****P* < 0.0001; HttQ89 vs HttQ89 + miR-7 (0.25 μg), ****P* = 0.0002; HttQ89 vs HttQ89 + miR-7 (0.5 μg), ****P* = 0.0003; HttQ89 vs HttQ89 + miR-7 (1.0 μg), ****P* = 0.0002;  4) **p-JNK**: Untransfected vs Untransfected + miR-7, *P* = 0.9987; Untransfected vs HttQ89, ****P* = 0.0004; HttQ89 vs HttQ89 + miR-7 (0.25 μg), ***P* = 0.0029; HttQ89 vs HttQ89 + miR-7 (0.5 μg), ***P* = 0.0030; HttQ89 vs HttQ89 + miR-7 (1.0 μg), ***P* = 0.0012;  5) **cleaved caspase-3**: Untransfected vs Untransfected + miR-7, *P* = 0.9846; Untransfected vs HttQ89, *****P* < 0.0001; HttQ89 vs HttQ89 + miR-7 (0.25 μg), *****P* < 0.0001; HttQ89 vs HttQ89 + miR-7 (0.5 μg), *****P* < 0.0001; HttQ89 vs HttQ89 + miR-7 (1.0 μg), *****P* < 0.0001.  **Fig. 4w** Statistical analysis was performed using one-way ANOVA followed by *post hoc* Tukey's test: Untransfected vs Untransfected + miR-7 (1.0 μg), *P* = 0.9904; Untransfected vs HttQ89, *****P* < 0.0001; HttQ89 vs HttQ89 + miR-7 (0.25 μg), ***P* = 0.0013; HttQ89 vs HttQ89 + miR-7 (0.5 μg), *****P* < 0.0001; HttQ89 vs HttQ89 + miR-7 (1.0 μg), *****P* < 0.0001. |
| **Fig. 5a** Statistical analysis was performed using one-way ANOVA followed by *post hoc* Tukey's test: Untransfected vs Untransfected + BCH001, *P* = 0.9991; Untransfected vs PAPD5, *****P* < 0.0001; PAPD5 vs PAPD5 + BCH001 (100 nM), ****P* = 0.0003; PAPD5 vs PAPD5 + BCH001 (200 nM), *****P* < 0.0001; PAPD5 vs PAPD5 + BCH001 (400 nM), *****P* < 0.0001.  **Fig. 5c** Statistical analysis was performed using one-way ANOVA followed by *post hoc* Tukey's test:  1) **p-TAK1**: Untransfected vs Untransfected + BCH001, *P* > 0.9999; Untransfected vs PAPD5, ****P* = 0.0002; PAPD5 vs PAPD5 + BCH001 (100 nM), ***P* = 0.0084; PAPD5 vs PAPD5 + BCH001 (200 nM), ***P* = 0.0051; PAPD5 vs PAPD5 + BCH001 (400 nM), ***P* = 0.0020;  2) **p-MKK4**: Statistical analysis was performed using one-way ANOVA followed by *post hoc* Tukey's test: Untransfected vs Untransfected + BCH001, *P* > 0.9999; Untransfected vs PAPD5, *****P* < 0.0001; PAPD5 vs PAPD5 + BCH001 (100 nM), *P* = 0.1594; PAPD5 vs PAPD5 + BCH001 (200 nM), ****P* = 0.0004; PAPD5 vs PAPD5 + BCH001 (400 nM), *****P* < 0.0001;  3) **p-JNK**: Statistical analysis was performed using one-way ANOVA followed by *post hoc* Tukey's test: Untransfected vs Untransfected + BCH001, *P* > 0.9999; Untransfected vs PAPD5, *****P* < 0.0001; PAPD5 vs PAPD5 + BCH001 (100 nM), **P* = 0.0106; PAPD5 vs PAPD5 + BCH001 (200 nM), ***P* = 0.0023; PAPD5 vs PAPD5 + BCH001 (400 nM), ****P* = 0.0007;  4) **cleaved caspase-3**: Statistical analysis was performed using one-way ANOVA followed by *post hoc* Tukey's test: Untransfected vs Untransfected + BCH001, *P* > 0.9999; Untransfected vs PAPD5, *****P* < 0.0001; PAPD5 vs PAPD5 + BCH001 (100 nM), ****P* = 0.0002; PAPD5 vs PAPD5 + BCH001 (200 nM), *****P* < 0.0001; PAPD5 vs PAPD5 + BCH001 (400 nM), *****P* < 0.0001.  **Fig. 5e** Statistical analysis was performed using one-way ANOVA followed by *post hoc* Tukey's test:  1) **p-TAK1**: Untransfected vs Untransfected + BCH001, *P* > 0.9999; HttQ23 vs HttQ23 + BCH001, *P* = 0.9994; HttQ89 vs HttQ89 + BCH001, *****P* < 0.0001; Untransfected vs HttQ23, *P* = 0.9972; HttQ23 vs HttQ89, *****P* < 0.0001;  2) **p-MKK4**: Untransfected vs Untransfected + BCH001, *P* > 0.9999; HttQ23 vs HttQ23 + BCH001, *P* = 0.9968; HttQ89 vs HttQ89 + BCH001, ****P* = 0.0002; Untransfected vs HttQ23, *P* > 0.9999; HttQ23 vs HttQ89, ****P* = 0.0001;  3) **p-JNK**: Untransfected vs Untransfected +U BCH001, *P* > 0.9999; HttQ23 vs HttQ23 + BCH001, *P* > 0.9999; HttQ89 vs HttQ89 + BCH001, ****P* = 0.0001; Untransfected vs HttQ23, *P* > 0.9999; HttQ23 vs HttQ89, *****P* < 0.0001;  4) **cleaved caspase-3**: Untransfected vs Untransfected + BCH001, *P* = 0.9931; HttQ23 vs HttQ23 + BCH001, *P* = 0.9992; HttQ89 vs HttQ89 + BCH001, *****P* < 0.0001; Untransfected vs HttQ23, *P* = 0.9988; HttQ23 vs HttQ89, *****P* < 0.0001.  **Fig. 5f** Statistical analysis was performed using one-way ANOVA followed by *post hoc* Tukey's test: Untransfected vs Untransfected + BCH001, *P* > 0.9999; HttQ23 vs HttQ23 + BCH001, *P* = 0.9975; HttQ89 vs HttQ89 + BCH001, *****P* < 0.0001; Untransfected vs HttQ23, **P* = 0.0385; HttQ23 vs HttQ89, *****P* < 0.0001.  **Fig. 5h** Statistical analysis was performed using two-tailed unpaired Student's *t*-test: HttQ93 vs HttQ93 + BCH001, ****P* = 0.0005. |
| **Fig. 6b** Statistical analysis was performed using two-tailed unpaired Student's *t*-test: wild-type vs YY1 mutant, *****P* < 0.0001.  **Fig. 6d-e** Statistical analysis was performed using two-tailed unpaired Student's *t*-test:  1) **YY1**: Control-siRNA vs YY1-siRNA, ****P* = 0.0003;  2) **PAPD5**: Control-siRNA vs YY1-siRNA, **P* = 0.0226.  **Fig. 6f-g** Statistical analysis was performed using two-tailed unpaired Student's *t*-test:  1) **dYY1**: GMR vs GMR + dYY1-dsRNA, ***P* = 0.0088;  2) **dPAPD5**: GMR vs GMR + dYY1-dsRNA, **P* = 0.0177.  **Fig. 6h** Statistical analysis was performed using one-way ANOVA followed by *post hoc* Tukey's test: CAG27 + wild-type vs CAG78 + wild-type, *****P* < 0.0001; CAG27 + YY1 mutant vs CAG78 + YY1 mutant, *P* = 0.0669.  **Fig. 6j** Statistical analysis was performed using one-way ANOVA followed by *post hoc* Tukey's test:  1) **PAPD5**: Untransfected + Control-siRNA vs Untransfected + YY1-siRNA, *P* = 0.2677; EGFPCAG27 + Control-siRNA vs EGFPCAG27 + YY1-siRNA, **P* = 0.0353; EGFPCAG78 + Control-siRNA vs EGFPCAG78 + YY1-siRNA, *****P* < 0.0001; Untransfected + Control-siRNA vs EGFPCAG27 + Control-siRNA, *P* = 0.9993; EGFPCAG27 + Control-siRNA vs EGFPCAG78 + Control-siRNA, *****P* < 0.0001;  2) **cleaved caspase-3**: Untransfected + Control-siRNA vs Untransfected + YY1-siRNA, **P* = 0.0467; EGFPCAG27 + Control-siRNA vs EGFPCAG27 + YY1-siRNA, *P* = 0.0544; EGFPCAG78 + Control-siRNA vs EGFPCAG78 + YY1-siRNA, ****P* = 0.0004; Untransfected + Control-siRNA vs EGFPCAG27 + Control-siRNA, *P* > 0.9999; EGFPCAG27 + Control-siRNA vs EGFPCAG78 + Control-siRNA, ****P* = 0.0002;  3) **YY1**: Untransfected + Control-siRNA vs Untransfected + YY1-siRNA, ****P* = 0.0006; EGFPCAG27 + Control-siRNA vs EGFPCAG27 + YY1-siRNA, ***P* = 0.0046; EGFPCAG78 + Control-siRNA vs EGFPCAG78 + YY1-siRNA, ***P* = 0.0076; Untransfected + Control-siRNA vs EGFPCAG27 + Control-siRNA, *P* = 0.6661; EGFPCAG27 + Control-siRNA vs EGFPCAG78 + Control-siRNA, *P* = 0.9991.  **Fig. 6l** Statistical analysis was performed using one-way ANOVA followed by *post hoc* Tukey's test:  1) **PAPD5**: Untransfected + Empty vector vs Untransfected + YY1, *P* > 0.9999; EGFPCAG27 + Empty vector vs EGFPCAG27 + YY1, *P* = 0.9997; EGFPCAG78 + Empty vector vs EGFPCAG78 + YY1, ***P* = 0.0012; Untransfected + Empty vector vs EGFPCAG27 + Empty vector, *P* = 0.9961; EGFPCAG27 + Empty vector vs EGFPCAG78 + Empty vector, *****P* < 0.0001;  2) **cleaved caspase-3**: Untransfected + Empty vector vs Untransfected + YY1, *P* = 0.9999; EGFPCAG27 + Empty vector vs EGFPCAG27 + YY1, *P* = 0.9942; EGFPCAG78 + Empty vector vs EGFPCAG78 + YY1, *****P* < 0.0001; Untransfected + Empty vector vs EGFPCAG27 + Empty vector, *P* = 0.9984; EGFPCAG27 + Empty vector vs EGFPCAG78 + Empty vector, *****P* < 0.0001;  3) **YY1**: Untransfected + Empty vector vs Untransfected + YY1, *****P* < 0.0001; EGFPCAG27 + Empty vector vs EGFPCAG27 + YY1, *****P* < 0.0001; EGFPCAG78 + Empty vector vs EGFPCAG78 + YY1, *****P* < 0.0001; Untransfected + Empty vector vs EGFPCAG27 + Empty vector, *P* > 0.9999; EGFPCAG27 + Empty vector vs EGFPCAG78 + Empty vector, *P* > 0.9999.  **Fig. 6n** Statistical analysis was performed using one-way ANOVA followed by *post hoc* Tukey's test:  1) **PAPD5**: Untransfected + Control-siRNA vs Untransfected + YY1-siRNA, *P* = 0.5638; HttQ23 + Control-siRNA vs HttQ23 + YY1-siRNA, *P* = 0.5770; HttQ89 + Control-siRNA vs HttQ89 + YY1-siRNA, **P* = 0.0157; Untransfected + Control-siRNA vs HttQ23 + Control-siRNA, *P* > 0.9999; HttQ23 + Control-siRNA vs HttQ89 + Control-siRNA, **P* = 0.0135;  2) **cleaved caspase-3**: Untransfected + Control-siRNA vs Untransfected + YY1-siRNA, *P* = 0.8665; HttQ23 + Control-siRNA vs HttQ23 + YY1-siRNA, *P* = 0.7040; HttQ89 + Control-siRNA vs HttQ89 + YY1-siRNA, ****P* = 0.0005; Untransfected + Control-siRNA vs HttQ23 + Control-siRNA, *P* > 0.9999; HttQ23 + Control-siRNA vs HttQ89 + Control-siRNA, ****P* = 0.0007;  3) **YY1**: Untransfected + Control-siRNA vs Untransfected + YY1-siRNA, *****P* < 0.0001; HttQ23 + Control-siRNA vs HttQ23 + YY1-siRNA, *****P* < 0.0001; HttQ89 + Control-siRNA vs HttQ89 + YY1-siRNA, **P* = 0.0116; Untransfected + Control-siRNA vs HttQ23 + Control-siRNA, *P* = 0.4603; HttQ23 + Control-siRNA vs HttQ89 + Control-siRNA, ****P* = 0.0006.  **Fig. 6p** Statistical analysis was performed using one-way ANOVA followed by *post hoc* Tukey's test:  1) **PAPD5**: Untransfected + Empty vector vs Untransfected + YY1, *P* = 0.9989; HttQ23 + Empty vector vs HttQ23 + YY1, *P* > 0.9999; HttQ89 + Empty vector vs HttQ89 + YY1, *****P* < 0.0001; Untransfected + Empty vector vs HttQ23 + Empty vector, *P* = 0.9716; HttQ23 + Empty vector vs HttQ89 + Empty vector, *****P* < 0.0001;  2) **cleaved caspase-3**: Untransfected + Empty vector vs Untransfected + YY1, *P* = 0.9987; HttQ23 + Empty vector vs HttQ23 + YY1, *P* = 0.9882; HttQ89 + Empty vector vs HttQ89 + YY1, *****P* < 0.0001; Untransfected + Empty vector vs HttQ23 + Empty vector, *P* > 0.9999; HttQ23 + Empty vector vs HttQ89 + Empty vector, *****P* < 0.0001;  3) **YY1**: Untransfected + Empty vector vs Untransfected + YY1, ****P* = 0.0001; HttQ23 + Empty vector vs HttQ23 + YY1, ****P* = 0.0001; HttQ89 + Empty vector vs HttQ89 + YY1, *****P* < 0.0001; Untransfected + Empty vector vs HttQ23 + Empty vector, *P* > 0.9999; HttQ23 + Empty vector vs HttQ89 + Empty vector, ****P* = 0.0003. |
| **Fig. 7a** Statistical analysis was performed using one-way ANOVA followed by *post hoc* Tukey's test: CAG0 vs CAG0 + dYY1-dsRNA, ****P* = 0.0005; CAG100 vs CAG100 + dYY1-dsRNA, ****P* = 0.0002; CAG0 vs CAG100, *P* = 0.5140.  **Fig. 7c** Statistical analysis was performed using one-way ANOVA followed by *post hoc* Tukey's test: CAG0 vs CAG0 + dYY1-dsRNA, ***P* = 0.0063; CAG0 vs CAG100, *****P* < 0.0001; CAG100 vs CAG100 + dYY1-dsRNA, *****P* < 0.0001.  **Fig. 7d** Statistical analysis was performed using one-way ANOVA followed by *post hoc* Tukey's test: GMR vs GMR + dYY1-dsRNA, ***P* = 0.0018; GMR > HttQ93 vs GMR > HttQ93 + dYY1-dsRNA, ***P* = 0.0031; GMR vs GMR > HttQ93, *P* = 0.8043.  **Fig. 7f** Statistical analysis was performed using one-way ANOVA followed by *post hoc* Tukey's test: GMR vs GMR + dYY1-dsRNA, *****P* < 0.0001; GMR vs GMR > HttQ93, *****P* < 0.0001; GMR > HttQ93 vs GMR > HttQ93 + dYY1-dsRNA, *****P* < 0.0001.  **Fig. 7g** Statistical analysis was performed using one-way ANOVA followed by *post hoc* Tukey's test: CAG0 vs CAG0 + dYY1, *****P* < 0.0001; CAG100 vs CAG100 + dYY1, *****P* < 0.0001; CAG0 vs CAG100, *P* = 0.9881.  **Fig. 7i** Statistical analysis was performed using one-way ANOVA followed by *post hoc* Tukey's test: CAG0 vs CAG0 + dYY1, *P* = 0.6744; CAG0 vs CAG100, *****P* < 0.0001; CAG100 vs CAG100 + dYY1, *****P* < 0.0001.  **Fig. 7j** Statistical analysis was performed using one-way ANOVA followed by *post hoc* Tukey's test: GMR vs GMR + dYY1, ****P* = 0.0001; GMR > HttQ93 vs GMR > HttQ93 + dYY1, *****P* < 0.0001; GMR vs GMR > HttQ93, *P* = 0.9993.  **Fig. 7l** Statistical analysis was performed using one-way ANOVA followed by *post hoc* Tukey's test: GMR vs GMR + dYY1, *P* = 0.7264; GMR vs GMR > HttQ93, *****P* < 0.0001; GMR > HttQ93 vs GMR > HttQ93 + dYY1, *****P* < 0.0001.  **Fig. 7n** Statistical analysis was performed using one-way ANOVA followed by *post hoc* Tukey's test: GMR > HttQ93 vs GMR > HttQ93 + dYY1, ****P* = 0.0002; GMR > HttQ93 vs GMR > HttQ93 + dPAPD5-dsRNA, ****P* = 0.0006; GMR > HttQ93 vs GMR > HttQ93 + dPAPD5-dsRNA + dYY1, ****P* = 0.0002; GMR > HttQ93 + dYY1 vs GMR > HttQ93 + dPAPD5-dsRNA + dYY1, *P* = 0.9797; GMR > HttQ93 + dPAPD5-dsRNA vs GMR > HttQ93 + dPAPD5-dsRNA + dYY1, *P* = 0.5545. |
| **Fig. 8a** Statistical analysis was performed using two-tailed unpaired Student's *t*-test: GMR vs GMR + HttQ93, **P* = 0.0145; CAG0 vs CAG100, *P* = 0.7361.  **Fig. 8d** Statistical analysis was performed using one-way ANOVA followed by *post hoc* Tukey's test:  1) **YY1**: Untransfected vs Q23(CAG), *P* > 0.9999; Q23(CAG) vs Q89(CAG), ***P* = 0.0022; Q89(CAG) vs CAG91, ***P* = 0.0016; Q89(CAG) vs Q89(CAA/G), *P* = 0.9563; CAG91 vs Q89(CAA/G), ****P* = 0.0007;  2) **PAPD5**: Untransfected vs Q23(CAG), *P* > 0.9999; Q23(CAG) vs Q89(CAG), *****P* < 0.0001; Q89(CAG) vs CAG91, ***P* = 0.0019; Q89(CAG) vs Q89(CAA/G), *****P* < 0.0001; CAG91 vs Q89(CAA/G), **P* = 0.0499.  **Fig. 8k** Statistical analysis was performed using one-way ANOVA followed by *post hoc* Tukey's test:  1) **TAB2**: Untransfected vs Q23(CAG), *P* = 0.9998; Q23(CAG) vs Q89(CAG), ****P* = 0.0004; Q89(CAG) vs CAG91, **P* = 0.0230; Q89(CAG) vs Q89(CAA/G), **P* = 0.0102; CAG91 vs Q89(CAA/G), *P* = 0.9817.  2) **p-TAK1**: Untransfected vs Q23(CAG), *P* = 0.9662; Q23(CAG) vs Q89(CAG), *****P* < 0.0001; Q89(CAG) vs CAG91, **P* = 0.0432; Q89(CAG) vs Q89(CAA/G), ***P* = 0.0012; CAG91 vs Q89(CAA/G), *P* = 0.1848.  3) **p-MKK4**: Untransfected vs Q23(CAG), *P* > 0.9999; Q23(CAG) vs Q89(CAG), ****P* = 0.0001; Q89(CAG) vs CAG91, **P* = 0.0160; Q89(CAG) vs Q89(CAA/G), **P* = 0.0139; CAG91 vs Q89(CAA/G), *P* > 0.9999.  4) **p-JNK**: Untransfected vs Q23(CAG), *P* > 0.9999; Q23(CAG) vs Q89(CAG), ****P* = 0.0002; Q89(CAG) vs CAG91, **P* = 0.0296; Q89(CAG) vs Q89(CAA/G), ***P* = 0.0076; CAG91 vs Q89(CAA/G), *P* = 0.8918.  5) **cleaved caspse-3**: Untransfected vs Q23(CAG), *P* = 0.9997; Q23(CAG) vs Q89(CAG), ****P* = 0.0008; Q89(CAG) vs CAG91, **P* = 0.0244; Q89(CAG) vs Q89(CAA/G), ***P* = 0.0050; CAG91 vs Q89(CAA/G), *P* = 0.8233.  **Fig. 8l** Statistical analysis was performed using one-way ANOVA followed by *post hoc* Tukey's test: Untransfected vs HttQ23(CAG), *P* = 0.9742; HttQ23(CAG) vs HttQ89(CAG), *****P* < 0.0001; HttQ89(CAG) vs HttCAG91, **P* = 0.0135; HttQ89(CAG) vs HttQ89(CAA/G), ****P* = 0.0001; HttCAG91 vs HttQ89(CAA/G), **P* = 0.0388. |
| **Fig. 9d** Statistical analysis was performed using one-tailed unpaired Student's *t*-test: Healthy control vs HD, **P* = 0.0277.  **Fig. 9f** Statistical analysis was performed using one-way ANOVA followed by *post hoc* Tukey's test:  1) **p-MKK4**: Healthy control vs Healthy control + BCH001, *P* = 0.8649; Healthy control vs HD, ***P* = 0.0020; HD vs HD + BCH001, **P* = 0.0120;  2) **p-JNK**: Healthy control vs Healthy control + BCH001, *P* = 0.8807; Healthy control vs HD, ***P* = 0.0043; HD vs HD + BCH001, **P* = 0.0296;  3) **cleaved caspase-3**: Healthy control vs Healthy control + BCH001, *P* = 0.9853; Healthy control vs HD, ****P* = 0.0002; HD vs HD + BCH001, ***P* = 0.0019.  **Fig. 9g** Statistical analysis was performed using one-tailed unpaired Student's *t*-test: Control vs HD, ***P* = 0.0067.  **Fig. 9i** Statistical analysis was performed using one-tailed unpaired Student's *t*-test: Control vs HD, *****P* < 0.0001.  **Fig. 9j** Statistical analysis was performed using one-tailed unpaired Student's *t*-test: Control vs HD, **P* = 0.0497.  **Fig. 9l** Statistical analysis was performed using one-tailed unpaired Student's *t*-test: Control vs HD, **P* = 0.0488.  **Fig. 9m** Statistical analysis was performed using two-tailed unpaired Student's *t*-test: Unaffected vs HD, ***P* = 0.0036;  **Fig. 9o** Statistical analysis was performed using two-tailed unpaired Student's *t*-test: Unaffected vs HD, ***P* = 0.0097;  **Fig. 9p** Statistical analysis was performed using two-tailed unpaired Student's *t*-test: Unaffected vs HD, *P* = 0.2086.  **Fig. 9r** Statistical analysis was performed using two-tailed unpaired Student's *t*-test: Unaffected vs HD, *P* = 0.1957. |
|  |
| **Supplementary Fig. 4c** Statistical analysis was performed using one-way ANOVA followed by *post hoc* Tukey's test: Untransfected vs Q23(CAG), ****P* = 0.0001; Untransfected vs Q89(CAG), ****P* = 0.0002; Untransfected vs CAG91, ****P* = 0.0002; Untransfected vs Q89(CAA/G), ****P* = 0.0002. |
| **Supplementary Fig. 6** Statistical analysis was performed using one-way ANOVA followed by *post hoc* Fisher's LSD test:  a) **let-7c-5p**: Untransfected vs PAPD5, ****P* = 0.0004; PAPD5 vs PAPD5^D256A & D258A^, ***P* = 0.0027, Untransfected vs PAPD5^D256A & D258A^, *P* = 0.0869;  b) **let-7g-5p**: Untransfected vs PAPD5, ****P* = 0.0001; PAPD5 vs PAPD5^D256A & D258A^, ****P* = 0.0003, Untransfected vs PAPD5^D256A & D258A^, *P* = 0.3006;  c) **let-7i-5p**: Untransfected vs PAPD5, ****P* = 0.0005; PAPD5 vs PAPD5^D256A & D258A^, ***P* = 0.0044, Untransfected vs PAPD5^D256A & D258A^, *P* = 0.0501;  d) **miR-10a-5p**: Untransfected vs PAPD5, **P* = 0.0274; PAPD5 vs PAPD5^D256A & D258A^, **P* = 0.0427, Untransfected vs PAPD5^D256A & D258A^, *P* = 0.7499;  e) **miR-101-3p**: Untransfected vs PAPD5, *****P* < 0.0001; PAPD5 vs PAPD5^D256A & D258A^, *****P* < 0.0001, Untransfected vs PAPD5^D256A & D258A^, *P* = 0.1543;  f) **miR-196a-5p**: Untransfected vs PAPD5, ***P* = 0.0020; PAPD5 vs PAPD5^D256A & D258A^, **P* = 0.0378, Untransfected vs PAPD5^D256A & D258A^, **P* = 0.0443;  g) **miR-28-3p**: Untransfected vs PAPD5, **P* = 0.0125; PAPD5 vs PAPD5^D256A & D258A^, **P* = 0.0222, Untransfected vs PAPD5^D256A & D258A^, *P* = 0.6611;  h) **miR-30d-5p**: Untransfected vs PAPD5, **P* = 0.0184; PAPD5 vs PAPD5^D256A & D258A^, **P* = 0.0383, Untransfected vs PAPD5^D256A & D258A^, *P* = 0.5921;  i) **miR-424-5p**: Untransfected vs PAPD5, ***P* = 0.0079; PAPD5 vs PAPD5^D256A & D258A^, **P* = 0.0126, Untransfected vs PAPD5^D256A & D258A^, *P* = 0.7058;  j) **miR-589-5p**: Untransfected vs PAPD5, ***P* = 0.0065; PAPD5 vs PAPD5^D256A & D258A^, ***P* = 0.0094, Untransfected vs PAPD5^D256A & D258A^, *P* = 0.7605. |
