# Supplementary material for: Mutant huntingtin induces neuronal apoptosis via derepressing the non-canonical poly(A) polymerase PAPD5
Source: Nat Commun. 2025 Apr 9;16:3307. doi: 10.1038/s41467-025-58618-4 (PMC11982267; doi:10.1038/s41467-025-58618-4)
Supplement: Supplementary file 2 — Description of Additional Supplementary Files [file 41467_2025_58618_MOESM2_ESM.pdf]

Description of Additional Supplementary Files

Supplementary Data 1. The list of 186 miRNAs manipulated by PAPD5 in EGFPCAG78-expressing SK-N-MC cells.

Supplementary Data 2. The adenylation level of processed miRNAs.

Supplementary Data 3. The list of genes that show reduced binding to YY1 in HD cell model.
