# Supplementary material for: Mutant huntingtin induces neuronal apoptosis via derepressing the non-canonical poly(A) polymerase PAPD5
Source: Nat Commun. 2025 Apr 9;16:3307. doi: 10.1038/s41467-025-58618-4 (PMC11982267; doi:10.1038/s41467-025-58618-4)
Supplement: Supplementary file 6 — Reporting Summary [file 41467_2025_58618_MOESM6_ESM.pdf]

nature portfolio

Corresponding author(s): Ho Yin Edwin Chan

Last updated by author(s): March 1, 2025

Reporting Summary

Nature Portfolio wishes to improve the reproducibility of the work that we publish. This form provides structure for consistency and transparency in reporting. For further information on Nature Portfolio policies, see our Editorial Policies and the Editorial Policy Checklist.

Statistics

For all statistical analyses, confirm that the following items are present in the figure legend, table legend, main text, or Methods section.

n/a	Confirmed	
☐	☒	The exact sample size (*n*) for each experimental group/condition, given as a discrete number and unit of measurement
☐	☒	A statement on whether measurements were taken from distinct samples or whether the same sample was measured repeatedly
☐	☒	The statistical test(s) used AND whether they are one- or two-sided<br>*Only common tests should be described solely by name; describe more complex techniques in the Methods section.*
☒	☐	A description of all covariates tested
☐	☒	A description of any assumptions or corrections, such as tests of normality and adjustment for multiple comparisons
☐	☒	A full description of the statistical parameters including central tendency (e.g. means) or other basic estimates (e.g. regression coefficient) AND variation (e.g. standard deviation) or associated estimates of uncertainty (e.g. confidence intervals)
☐	☒	For null hypothesis testing, the test statistic (e.g. *F*, *t*, *r*) with confidence intervals, effect sizes, degrees of freedom and *P* value noted<br>*Give P values as exact values whenever suitable.*
☒	☐	For Bayesian analysis, information on the choice of priors and Markov chain Monte Carlo settings
☒	☐	For hierarchical and complex designs, identification of the appropriate level for tests and full reporting of outcomes
☐	☒	Estimates of effect sizes (e.g. Cohen's *d*, Pearson's *r*), indicating how they were calculated

Our web collection on statistics for biologists contains articles on many of the points above.

Software and code

Policy information about availability of computer code

Data collection	All blots were visualized using the Bio-rad ChemiDoc Touch Imaging System;<br>Thermal cycling was performed on a Bio-Rad CFX96 Real-time PCR detection system;<br>Confocal images were acquired on a Leica TCS SP8 high speed imaging system;<br>All absorbance and luminescence readings were recorded on a Spark® multimode microplate reader.
Data analysis	The intensities of the protein bands were quantified using the ImageJ software version 1.52k;<br>Confocal images were analyzed using the Leica Application Suite X software;<br>All absorbance and luminescence readings were determined by the SparkControl software version 2.1;<br>GraphPad Prism version 10.0.3 was used to plot data and perform statistical analysis;<br>The illustrations were created using Adobe Illustrator 27.0.1 (Fig. 1m, 1p, 3c, 3k and 6a) and BioRender.com (Fig. 8b, 10 and Supplementary Fig. 5a). Assembly of panels in finalized figures was done using Adobe Illustrator 27.0.1.<br><br>Any codes used for the small RNA-seq and ChIP-seq data analyses are available upon reasonable request to the corresponding author (hyechan@cuhk.edu.hk).

For manuscripts utilizing custom algorithms or software that are central to the research but not yet described in published literature, software must be made available to editors and reviewers. We strongly encourage code deposition in a community repository (e.g. GitHub). See the Nature Portfolio guidelines for submitting code & software for further information.

Data

Policy information about availability of data

All manuscripts must include a data availability statement. This statement should provide the following information, where applicable:

- Accession codes, unique identifiers, or web links for publicly available datasets
- A description of any restrictions on data availability
- For clinical datasets or third party data, please ensure that the statement adheres to our policy

Transcription factor binding sites were predicted using Transcription factor affinity prediction (http://trap.molgen.mpg.de/cgi-bin/trap_form.cgi),92 PROMO (http://alggen.lsi.upc.es/cgi-bin/promo_v3/promo/promoinit.cgi?dirDB=TF_8.3.)93 and JASPAR (http://jaspar.genereg.net/)94 databases. The human, rat, mouse, and fly PAPD5 promoter sequences were withdrawn from GenBank under accession codes NC_000016.10 (https://www.ncbi.nlm.nih.gov/nuccore/NC_000016.10?from=50152911&to=50235310&report=genbank), NC_086037.1 (https://www.ncbi.nlm.nih.gov/nuccore/NC_086037.1?from=34984244&to=35042423&report=genbank&strand=true), NC_000074.7 (https://www.ncbi.nlm.nih.gov/nuccore/NC_000074.7?from=88925229&to=88989942&report=genbank), and NT_033777.3 (https://www.ncbi.nlm.nih.gov/nuccore/NT_033777.3?from=24925216&to=24927085&report=genbank), respectively. The raw cycle threshold values for miRNA array have been deposited in GEO under accession code GSE271666 (https://www.ncbi.nlm.nih.gov/geo/query/acc.cgi?acc=GSE271666). The raw sequencing files for small RNA-seq and ChIP-seq have been deposited in GEO under accession codes GSE272903 (https://www.ncbi.nlm.nih.gov/geo/query/acc.cgi?acc=GSE272903) and GSE273082 (https://www.ncbi.nlm.nih.gov/geo/query/acc.cgi?acc=GSE273082), respectively. All data supporting the findings of this study are presented in the main manuscript and as Supplementary Information. Source data are provided with this paper.

Research involving human participants, their data, or biological material

Policy information about studies with human participants or human data. See also policy information about sex, gender (identity/presentation), and sexual orientation and race, ethnicity and racism.

Reporting on sex and gender	We obtained three iPSC lines from healthy individuals (2 female and 1 male) and three iPSC lines from HD patients (3 female). We have also obtained brain samples (striatum and cerebellum) from seven unaffected controls (1 female and 6 male) and seven HD patients (3 female and 4 male). No sex-disaggregated analysis was performed.
Reporting on race, ethnicity, or other socially relevant groupings	Data on race, ethnicity, or other socially relevant groupings is not considered in this study.
Population characteristics	The post-mortem striatal and cerebellar tissues were collected from 7 HD patients and 7 unaffected individuals, with age ranges from 22 to 78 years. The iPSCs were from 3 HD patients and 3 healthy controls, with age ranges from 20 to 78 years.
Recruitment	The unaffected individuals and HD patients were enrolled by the Neurological Foundation Human Brain Bank without selection-bias. There is also no selection-bias for choosing iPSC lines.
Ethics oversight	Post-mortem patient tissues were provided by the Neurological Foundation Human Brain Bank with institutional ethics approval #011654 (7 HD patients and 7 unaffected individuals; striatum and cerebellum) directed by Richard L.M. Faull and Maurice A. Curtis. The healthy control iPSCs were derived from human skin biopsy fibroblasts, collected under ethical approval granted by the South Wales Research Ethics Committee (WA/12/0186) in the James Martin Stem Cell Facility, University of Oxford, under standardized protocols. The HD iPSCs were obtained from NINDS Human Cell and Data Repository.

Note that full information on the approval of the study protocol must also be provided in the manuscript.

Field-specific reporting

Please select the one below that is the best fit for your research. If you are not sure, read the appropriate sections before making your selection.

☒ Life sciences ☐ Behavioural & social sciences ☐ Ecological, evolutionary & environmental sciences

For a reference copy of the document with all sections, see nature.com/documents/nr-reporting-summary-flat.pdf

Life sciences study design

All studies must disclose on these points even when the disclosure is negative.

Sample size	No sample size calculation was performed for this work. The number of iPSC lines/differentiation replicates used in this study represents common practice in the field (Arber et al., 2015, Development). Unless specified otherwise n = 3 biologically independent experiments. The data met the assumptions for each statistical test performed.
Data exclusions	No data exclusions was done.
Replication	Experimental data were collected from at least three independent trials. Number of biological replicates of cell- or animal-based experiments were described in the figure legends of the manuscript.

Randomization	The cells and animals were randomly assigned for experiments.
Blinding	Investigators who conduct the experiments were blinded to group allocation during data collection and analysis.

Reporting for specific materials, systems and methods

We require information from authors about some types of materials, experimental systems and methods used in many studies. Here, indicate whether each material, system or method listed is relevant to your study. If you are not sure if a list item applies to your research, read the appropriate section before selecting a response.

Materials & experimental systems

n/a	Involved in the study
☐	☒ Antibodies
☐	☒ Eukaryotic cell lines
☒	☐ Palaeontology and archaeology
☐	☒ Animals and other organisms
☒	☐ Clinical data
☒	☐ Dual use research of concern
☒	☐ Plants

Methods

n/a	Involved in the study
☐	☒ ChIP-seq
☒	☐ Flow cytometry
☒	☐ MRI-based neuroimaging

Antibodies

Antibodies used

For immunocytochemistry performed on SK-N-MC cells, the primary and secondary antibodies used were anti-YY1 (1:200, ab109237, Abcam), anti-polyglutamine (1:200, MAB1574, Merck Millipore), Alexa Fluor 488 Donkey anti-Rabbit IgG (H+L) (1:500, ab150073, Abcam) and Alexa Fluor 594 Donkey anti-Mouse IgG (H+L) (1:500, A-21203, Thermo Fisher Scientific).

For immunocytochemistry performed on iPSC-derived striatal neurons, the primary antibodies used were anti-YY1 (1:200, ab109237, Abcam), anti-DARPP32 (1:200, ab40801, Abcam), anti-polyglutamine (1:200, MAB1574, Merck Millipore) and anti-MAP2 (1:10,000, ab5392, Abcam). Secondary antibodies used were Alexa Fluor 488 Donkey anti-Rabbit IgG (H+L) (1:500, ab150073, Abcam), Alexa Fluor 594 Donkey anti-Mouse IgG (H+L) (1:500, A-21203, Thermo Fisher Scientific) and Alexa Fluor 647 Goat anti-Chicken IgY (H+L) (1:500, ab150171, Abcam).

For immunoblotting performed on SK-N-MC cells and iPSC-derived striatal neurons, primary antibodies used were anti-PAPD5 (PA5-46747, 1:1,000) from Invitrogen; anti-p-TAK1 (4536, 1:1,000), anti-t-TAK1 (4505, 1:2,000), anti-p-MKK4 (9156, 1:1,000), anti-t-MKK4 (9152, 1:2,000), anti-p-JNK (9251, 1:2,000), anti-t-JNK (9252, 1:2,000), anti-TAB2 (3745, 1:2,000), anti-cleaved caspase-3 (9664, 1:500) and anti-myc (2276, 1:2,000) from Cell Signaling Technology; anti-Huntingtin (MAB5374, 1:1,000) from Merck Millipore; anti-HA (H3663, 1:2,000) from Sigma-Aldrich; anti-YY1 (ab109237, 1:2000) and anti-β-tubulin/dβ-tubulin (ab6046, 1:2,000) from Abcam; anti-GFP (632381, 1:4,000) from Clontech, and anti-dYY1 (orb806899, 1:1,000) from Biorbyt. Secondary antibodies used were goat anti-rabbit (11-035-045, 1:5,000) and goat anti-mouse (115-035-062, 1:5,000) from Jackson ImmunoResearch.

For immunoblotting performed on human brain samples, the primary antibodies used were anti-PAPD5 (1:1,000, 55197-1-AP, Proteintech), anti-TAB2 (1:1,000, 3745, Cell Signaling Technology) and anti-Actin (1:30,000, 612657, BD Transduction Laboratory).

Validation

The anti-PAPD5 antibody (PA5-46747, Thermo Fisher Scientific) detects human PAPD5 protein and is suitable for WB (https://www.thermofisher.com/antibody/product/PAPD5-Antibody-Polyclonal/PA5-46747);
The anti-PAPD5 antibody (55197-1-AP, Proteintech) detects human PAPD5 protein and is suitable for WB (https://www.ptglab.com/products/PAPD5-Antibody-55197-1-AP.htm);
The anti-p-TAK1 antibody (4536, Cell Signaling Technology) detects human p-TAK1 protein and is suitable for WB (https://www.cellsignal.com/products/primary-antibodies/phospho-tak1-thr187-antibody/4536);
The anti-t-TAK1 antibody (4505, Cell Signaling Technology) detects human t-TAK1 protein and is suitable for WB (https://www.cellsignal.com/products/primary-antibodies/tak1-antibody/4505);
The anti-p-MKK4 antibody (9156, Cell Signaling Technology) detects human p-MKK4 protein and is suitable for WB (https://www.cellsignal.com/products/primary-antibodies/phospho-sek1-mkk4-ser257-thr261-antibody/9156);
The anti-t-MKK4 antibody (9152, Cell Signaling Technology) detects human t-MKK4 protein and is suitable for WB (https://www.cellsignal.com/products/primary-antibodies/sek1-mkk4-antibody/9152);
The anti-p-JNK antibody (9251, Cell Signaling Technology) detects human p-JNK proteins and is suitable for WB (https://www.cellsignal.com/products/primary-antibodies/phospho-sapk-jnk-thr183-tyr185-antibody/9251);
The anti-t-JNK antibody (9252, Cell Signaling Technology) detects human t-JNK proteins and is suitable for WB (https://www.cellsignal.com/products/primary-antibodies/sapk-jnk-antibody/9252);
The anti-TAB2 antibody (3745, Cell Signaling Technology) detects human t-TAB2 protein and is suitable for WB (https://www.cellsignal.com/products/primary-antibodies/tab2-c88h10-rabbit-mab/3745);
The anti-cleaved caspase 3 (9664, Cell Signaling Technology) detects human cleaved caspase 3 protein and is suitable for WB (https://www.cellsignal.com/products/primary-antibodies/cleaved-caspase-3-asp175-5a1e-rabbit-mab/9664);
The anti-myc (2276, Cell Signaling Technology) detects myc-tagged proteins and is suitable for WB (https://www.cellsignal.com/products/primary-antibodies/myc-tag-9b11-mouse-mab/2276);
The anti-Huntingtin (MAB5374, Merck Millipore) detects human Huntingtin protein and is suitable for WB (https://www.merckmillipore.com/HK/en/product/Anti-Huntingtin-Protein-Antibody-clone-mEM48,MM_NF-MAB5374);
The anti-YY1 antibody (ab109237, Abcam) detects human YY1 protein and is suitable for WB and ICC (https://www.abcam.com/

products/primary-antibodies/yy1-antibody-epr4652-nuclear-loading-control-ab109237.html);
The anti-dYY1 antibody (orb806899, Biorbyt) detects fly dYY1 protein and is suitable for WB (https://www.biorbyt.com/pho-antibody-orb806899.html);
The anti-β-tubulin/dβ-tubulin antibody (ab6046, Abcam) detects human β-tubulin protein and is suitable for WB (https://www.abcam.com/products/primary-antibodies/beta-tubulin-antibody-loading-control-ab6046.html), and we have demonstrated in our paper that it detects fly β-tubulin protein at correct size;
The anti-Actin antibody (612657, BD Transduction Laboratory) detect human Actin protein and is suitable for WB (https://www.bdbiosciences.com/en-eu/products/reagents/microscopy-imaging-reagents/immunofluorescence-reagents/purified-mouse-anti-actin-ab-5.612657);
The anti-GFP antibody (632381, Takara) detects GFP-tagged proteins and is suitable for WB (https://www.takarabio.com/products/antibodies-and-elisa/fluorescent-protein-antibodies/green-fluorescent-protein-antibodies);
The anti-polyglutamine antibody (MAB1574, Merck Millipore) detects expanded polyglutamine-containing proteins and is suitable for ICC (https://www.merckmillipore.com/HK/en/product/Anti-Polyglutamine-Expansion-Diseases-Marker-Antibody-clone-5TF1-1C2,MM_NF-MAB1574);
The anti-DARPP32 antibody (ab40801, Abcam) detects human DARPP32 protein and is suitable for ICC (https://www.abcam.com/en-us/products/primary-antibodies/darpp32-antibody-ep720y-ab40801);
The anti-MAP2 antibody (ab5392, Abcam) detects human MAP2 protein and is suitable for ICC (https://www.abcam.com/products/primary-antibodies/map2-antibody-ab5392.html).

Eukaryotic cell lines

Policy information about cell lines and Sex and Gender in Research

Cell line source(s)	SK-N-MC cells, ATCC®, HTB-10TM;<br>The three healthy control iPSC lines were derived from human skin biopsy fibroblasts, collected under ethical approval granted by the South Wales Research Ethics Committee (WA/12/0186) in the James Martin Stem Cell Facility, University of Oxford, under standardized protocols. The line IDs are SFC-840-03-03, SFC-841-03-01 and SFC-856-03-04;<br>The HD iPSCs were obtained from NINDS Human Cell and Data Repository. The line IDs are ND36998, ND41656 and ND42230.
Authentication	The SK-N-MC cell line is available and authenticated at ATCC;<br>All healthy control iPSCs were generated in-house and have been published and characterised extensively before (Ababneh et al., 2020, Hum Mol Genet; Dafinca et al., 2020, Stem Cell Rep; Vahsen et al., 2023, Nat Commun);<br>The HD iPSC lines obtained from Coriell were authenticated at NIGMS Human Genetic Cell Repository.
Mycoplasma contamination	Cells were tested negative for mycoplasma contamination.
Commonly misidentified lines<br>(See ICLAC register)	Despite being listed as a misidentified cell line (sarcoma instead of neuroblastoma; Registration ID: ICLAC-00267), our SK-N-MC cell line is obtained from a different commercial source (ATCC, USA) as the previously misidentified SK-N-MC cells (Deutsche Sammlung für Zellkulturen und Mikroorganismen; Germany). Our SK-N-MC cell line is of neurogenic origin as stated by ATCC (https://www.atcc.org/products/htb-10).

Animals and other research organisms

Policy information about studies involving animals; ARRIVE guidelines recommended for reporting animal research, and Sex and Gender in Research

Laboratory animals	Fly lines GMR-GAL4 (Ellis et al., 1993, Development), UAS-DsRedCAG0/100 (Li et al., 2008, Nature), UAS-Httexon1Q93 (Steffan et al., 2001, Nature), UAS-CTG60/480 (Garcia-Lopez et al., 2008, PLoS One) and UAS-CGG90-EGFP (Jin et al., 2003, Neuron) were described previously. The UAS-PAPD5 dsRNA line (GD19799) and UAS-dYY1 dsRNA line (GD39529) were obtained from Vienna Drosophila RNAi Center. The UAS-dYY1 line (F000151) and Tub56DGene-Switch-GAL4 line (40333) were obtained from FlyORF and Bloomington Drosophila Stock Center, respectively. All flies were maintained in cornmeal culture medium and genetic crosses were set up in a 21.5C incubator (LMS).
Wild animals	No wild animals were involved in this study.
Reporting on sex	Both male and female flies were used in this study.
Field-collected samples	No field-collected samples were involved in this study.
Ethics oversight	All animal procedures were approved by the CUHK Animal Experimentation Ethics Committee (and their care was in accordance with the institutional guidelines).

Note that full information on the approval of the study protocol must also be provided in the manuscript.

Plants

Seed stocks	N/A
Novel plant genotypes	N/A
Authentication	N/A

ChIP-seq

Data deposition

☒ Confirm that both raw and final processed data have been deposited in a public database such as GEO.

☐ Confirm that you have deposited or provided access to graph files (e.g. BED files) for the called peaks.

Data access links<br>*May remain private before publication.*	https://www.ncbi.nlm.nih.gov/geo/query/acc.cgi?acc=GSE273082
Files in database submission	GSM8421577 SK-N-MC cells, Htt1-550Q23, biol rep 1<br>GSM8421578 SK-N-MC cells, Htt1-550Q89, biol rep 1<br>GSM8421579 SK-N-MC cells, Htt1-550Q23, biol rep 2<br>GSM8421580 SK-N-MC cells, Htt1-550Q89, biol rep 2<br>GSM8421581 SK-N-MC cells, Htt1-550Q23, biol rep 3<br>GSM8421582 SK-N-MC cells, Htt1-550Q89, biol rep 3<br>GSM8421583 SK-N-MC cells, Input control, biol rep 1<br>GSM8421584 SK-N-MC cells, Input control, biol rep 2<br>GSM8421585 SK-N-MC cells, Input control, biol rep 3
Genome browser session<br>(e.g. UCSC)	N/A

Methodology

Replicates	Three independent biological replicates were performed.
Sequencing depth	DNA libraries were sequenced at paired-end PE150 in the Illumina NovaSeq 6000.
Antibodies	The anti-YY1 antibody (22156-1-AP, Proteintech) detects human YY1 protein and is suitable for ChIP (https://www.ptglab.com/products/YY1-Antibody-22156-1-AP.htm).
Peak calling parameters	The YY1-binding DNA sites (peaks) were identified using MACS3 v3.0.1 with input control as background, annotated using ChIPseeker v1.38.0, and visualized using Trackplot v1.5.10.
Data quality	Raw sequencing files were checked using FastQC v0.11.8. Adapter and quality trimming were performed using Cutadapt v3.7. FastQC was performed on the trimmed data again to confirm the removal of adapter sequence. The reads were then aligned to human genome GRCh38.p14 using Bowtie2 v2.4.1. Uniquely aligned reads were retrieved using Sambamba v1.0.1, and those overlapping with regions in the Encyclopedia of DNA Elements (ENCODE) blacklist were removed using BEDTools v2.29.2.
Software	Raw sequencing files were checked using FastQC v0.11.8. Adapter and quality trimming were performed using Cutadapt v3.7. FastQC was performed on the trimmed data again to confirm the removal of adapter sequence. The reads were then aligned to human genome GRCh38.p14 using Bowtie2 v2.4.1. Uniquely aligned reads were retrieved using Sambamba v1.0.1, and those overlapping with regions in the Encyclopedia of DNA Elements (ENCODE) blacklist were removed using BEDTools v2.29.2. The YY1-binding DNA sites (peaks) were identified using MACS3 v3.0.1 with input control as background, annotated using ChIPseeker v1.38.0, and visualized using Trackplot v1.5.10. Read counts were obtained following the normalization to sequencing depth using DiffBind v3.12.0. The peaks with read counts more than zero in each of the samples were further processed. Promoter was defined as ± 2 kilobases from a gene’s transcription start site (TSS). The YY1-targeted genes were selected according to the ENCODE project. The YY1-targeted genes that show reduced binding to YY1 in EGFP-Htt1-550Q89-expressing cells are listed in Supplementary Data 3. Statistical analysis was performed using one-tailed unpaired Student's t-test.
